# Integrating the hallmarks of cancer into autophagy: a perspective from underlying mechanisms to therapeutic strategies

**DOI:** 10.7150/thno.135134

**Published:** 2026-05-18

**Authors:** Huidi Liu, Wei Liu, Xiaochun Zhang, Yufeng Jiang, Heng Xu, Ningning Wang, Bo Liu, Na Lin

**Affiliations:** 1 Department of Anus and Intestine Surgery, The First Hospital of China Medical University, Shenyang 110001, China.; 2 Department of Biotherapy, Cancer Center and State Key Laboratory of Biotherapy, West China Hospital, Sichuan University, Chengdu 610041, China.; 3 Department of Respiratory and Critical Care Medicine, The First Hospital of China Medical University, Shenyang 110001, China.; 4 Department of Emergency, The First Hospital of China Medical University, Shenyang 110001, China.; 5 Department of Gastroenterology, The First Hospital of China Medical University, Shenyang 110001, China.; 6 Department of Hematology, The First Hospital of China Medical University, Shenyang 110001, China.

**Keywords:** autophagy, cancer hallmarks, autophagy modulators, crosstalk, target therapy, molecular mechanism

## Abstract

Despite remarkable advances in cancer therapy, clinical outcomes remain limited by drug resistance, metastasis, and off-target effects that stem from the complexity and heterogeneity of tumors. The “hallmarks of cancer” provides a systematic framework for understanding tumor biology and identifying therapeutic targets. However, the expression patterns and mechanistic dependencies of these hallmarks differ markedly among cancer types. Autophagy is an evolutionarily conserved catabolic process, exerting multifaceted and context-dependent functions in tumor initiation and malignant progression. In this review, we summarize current insights into the regulation of autophagy and its impact on key signaling pathways. Most importantly, based on the characteristics of tumor progression, we classified the 14 hallmarks of cancer into four categories and discussed the crosstalk between autophagy and these hallmarks. In addition, we survey recent progress in the discovery of small-molecule compounds targeting autophagy and evaluate their therapeutic implications from a hallmark-oriented perspective. Finally, this review highlight that integrating the conceptual framework of cancer hallmarks with the biological and pharmacological functions of autophagy offers a promising avenue for precision oncology. Elucidating how autophagy differentially modulates distinct hallmarks, as synthesized in this review, will be instrumental in facilitating context-specific interventions and guiding future strategies for personalized cancer therapy.

## Introduction

Cancer remains a profound global health crisis and a formidable economic burden, with forecasts suggesting that this challenge will escalate significantly in the years ahead 1. While substantial progress has undeniably been made in developing cancer treatments, significant hurdles continue to impede consistently successful therapeutic outcomes. A key factor complicating treatment is the intrinsic heterogeneity of the cancer itself 2. Tumorigenesis is a multistep process driven by cumulative genetic alterations that transform normal human cells into highly malignant derivatives. The pathways that cells undertake during malignant progression are highly heterogeneous, conferring substantial tumor heterogeneity and diverse biological capabilities. Therefore, delineating the hallmark capabilities of cancer is essential for both understanding tumor biology and developing effective therapeutic strategies. The conceptualization of cancer hallmarks originated from the foundational work of Douglas Hanahan and Robert Weinberg 3. Their seminal 2000 publication established six core capabilities that are universally acquired during cancer development: sustained proliferative signaling independent of external growth factors, evasion of growth-suppressive mechanisms, resistance to programmed cell death, limited replicative potential through telomere maintenance, induction of blood vessel formation termed angiogenesis, and activation of tissue invasion coupled with metastasis 4. This framework provides a unifying paradigm for understanding malignant transformation. In 2011, the authors expanded this model to ten hallmarks by incorporating two enabling characteristics and two additional hallmarks. The enabling characteristics include genome instability, which generates mutational diversity, and tumor-promoting inflammation, which fosters a permissive microenvironment. The newly added hallmarks are metabolic reprogramming, in which cancer cells preferentially undergo aerobic glycolysis even under oxygen-rich conditions, and the ability to avoid immune destruction 5. Their 2022 update further introduced emerging dimensions, including unlocking phenotypic plasticity that enables cellular differentiation and transdifferentiation, nonmutational epigenetic reprogramming that alters gene expression without DNA sequence changes, polymorphic microbial influences within the tumor ecosystem, and the functional impact of senescent cells on the tissue microenvironment. The delineation of these hallmarks provides a comprehensive logical framework for understanding tumorigenesis and tumor progression while also offering critical insights for therapeutic development 6. In the latest 2026 update, the hallmarks of cancer are summarized through a four-dimensional framework: the first dimension comprises the nine core hallmarks (acquired functional capabilities); the second encompasses five enabling characteristics (phenotypic traits); the third consists of the cellular constituents that form the tumor microenvironment; and the fourth addresses the interactions between cancer, as a systemic disease, and the host organism 7.

Autophagy is an umbrella term encompassing all the cellular pathways that deliver cytoplasmic constituents to lysosomes in animal cells or vacuoles in plant and fungal cells 6. This process can be broadly classified into three principal categories: macroautophagy, microautophagy, and chaperone-mediated autophagy (CMA) 8. Among these autophagic pathways, macroautophagy represents the predominant and most extensively characterized mechanism, significantly overshadowing microautophagy and CMA as research foci. Macroautophagy plays a complex and context-dependent role in cancer, functioning as a double-edged sword that can either suppress tumor initiation or promote tumor progression and therapy resistance 9. Autophagy intersects with multiple cancer hallmarks to regulate the progression of cancer. Autophagy directly contributes to sustained proliferative signaling by degrading negative regulators, such as Phosphatase and Tensin Homolog (PTEN) and Tumor Protein 53 (p53), thereby enhancing oncogenic signaling 10. It enables the evasion of growth suppressors via the autophagic clearance of cell cycle inhibitors such as p27. It facilitates resistance to cell death by reducing reactive oxygen species (ROS)-induced damage via mitophagy 11. Furthermore, autophagy supports replicative immortality by maintaining telomere stability and cancer stem cell function. It promotes angiogenesis through hypoxia-induced HIF-1α activation and vascular endothelial growth factor (VEGF) secretion and drives invasion and metastasis by facilitating epithelial‒mesenchymal transition (EMT) and the release of prometastatic factors such as matrix metalloproteinases (MMPs) and IL-6 12. Metabolic reprogramming, a key emerging hallmark, relies heavily on autophagy to recycle nutrients, including amino acids and lipids, thereby supporting energy and biomass generation under nutrient scarcity. The activation of autophagy by the USP19/NEK9 axis potentiates autophagic cell death by inhibiting the Warburg effect in pancreatic cancer 13. Additionally, autophagy helps tumors evade immune destruction by modulating the expression of antigens and immune checkpoints, such as PD-L1. It contributes to tumor-promoting inflammation by regulating cytokine release and inflammasome activity 14. It also maintains genomic stability by limiting DNA damage and facilitates nonmutational epigenetic reprogramming by degrading histone modifiers and providing metabolic cofactors 15. These multifaceted interactions illustrate how autophagy acts as a keystone process enabling tumors to adapt, survive, and resist therapies. The strategic inhibition or induction of autophagy, depending on the tumor type, stage, and hallmark vulnerability, offers a promising approach to overcome drug resistance and inhibit tumor progression.

In this review, we reconceptualize the 2022 cancer hallmarks framework from a mechanistic perspective and propose a classification into four categories based on tumor initiation and progression: proliferative hallmarks, dissemination hallmarks, stress and plasticity hallmarks, and microenvironmental and immune hallmarks. We explain the rationale for this categorization and systematically describe the crosstalk between autophagy and each hallmark category. In addition, we review the molecular mechanisms by which small-molecule autophagy modulators regulate these cancer hallmarks, offering a comprehensive perspective for the development of novel autophagy-based therapeutic strategies. By bridging the cancer hallmarks theory with autophagy biology, this review offers a novel conceptual framework that deepens our understanding of cancer pathogenesis and provides a comprehensive perspective essential for the development of more effective and individualized autophagy-based therapeutic strategies.

## 1. Molecular Mechanisms and Regulation of Autophagy

Autophagy is a conserved lysosomal degradation process orchestrated by a hierarchical signaling cascade. It initiates with the sequestration of cytoplasmic material by an expanding phagophore 16, a process regulated by the ULK1 kinase complex (ULK1/2, FIP200, ATG13) 17-19 and the Class III PI3K complex (VPS34, ATG14, Beclin1, UVRAG) 20. Under nutrient-replete conditions, mTORC1 suppresses autophagy by phosphorylating ULK1; conversely, nutrient deprivation inactivates mTORC1 to trigger the ULK1-PI3K activation cascade 21,22. The subsequent nucleation at the phagophore assembly site (PAS) and membrane elongation are driven by ATG9A-positive vesicles and two ubiquitin-like conjugation systems: the ATG12-ATG5 and ATG8-LC3 pathways 23-25. These systems facilitate cargo sequestration and the closure of the mature autophagosome. Finally, autophagosomes are actively transported to the perinuclear region to fuse with lysosomes, where lysosomal enzymes degrade the sequestered material to recycle nutrients for cellular reuse 26.

Autophagy regulation is a multi-layered process integrating transcriptional, post-transcriptional, and environmental signals 27. We first focus on the autophagic response triggered by nutrient deprivation, as it is the most widely characterized physiological inducer of autophagy. The target of rapamycin (TOR) kinase has long been established as the central regulator of this process. TORC1 coordinates a broad transcriptional program in response to starvation 20. Under nutrient-rich conditions, mTORC1 phosphorylates the Transcription Factor EB (TFEB), the master regulator of autophagy and lysosomal biogenesis. This mTORC1-mediated phosphorylation at Ser211 induces TFEB binding to 14-3-3 proteins, leading to its cytoplasmic sequestration. Conversely, during nutrient scarcity or energy stress, mTORC1 activity is suppressed, allowing TFEB to undergo dephosphorylation and rapidly translocate into the nucleus. Once in the nucleus, TFEB binds to Coordinated Lysosomal Expression and Regulation (CLEAR) elements within the promoters of autophagy-related genes, orchestrating a comprehensive transcriptional program that upregulates the entire autophagic machinery, from initiation to lysosomal degradation 28. Next, environmental stressors precisely regulate autophagy through dedicated molecular pathways 23. Hypoxia stabilizes hypoxia-inducible factor 1α (HIF-1α) by inhibiting oxygen-sensing prolyl hydroxylases (PHDs). HIF-1α transcriptionally upregulates the mitophagy receptors BNIP3, NIX, and FUNDC1, which recruit autophagosomes via LC3-interacting regions (LIRs) to clear damaged mitochondria 29. This pathway maintains cellular homeostasis during oxygen deprivation but contributes to cancer progression when dysregulated. Endoplasmic reticulum (ER) stress activates the unfolded protein response (UPR), where the IRE1α kinase splices XBP1 mRNA to generate the transcription factor XBP1s 30. XBP1s directly induces the expression of autophagy genes and cooperates with ROS-dependent PTEN-induced putative kinase 1 (PINK1) and E3 ubiquitin ligase Parkin (PINK1/Parkin) activation to promote organelle-selective autophagy 31. Moreover, some specific downstream transcriptional targets directly responsible for modulating autophagy have remained poorly defined until recently 32. Emerging evidence has begun to delineate a network of transcription factors dedicated to autophagy regulation, with key factors such as TP53, STAT3, and NF-κB exhibiting dual functions as both activators and repressors. For instance, nuclear p53 promotes autophagy by directly targeting the TFEB promoter or inducing DRAM1 expression, whereas cytoplasmic p53 serves as a potent inhibitor by interacting with the ULK1 complex 33. This bifunctionality is achieved through distinct mechanisms, whereby they regulate transcription via nuclear interactions and modulate autophagy independently of transcription through cytoplasmic signaling 34.

**Figure 1:**
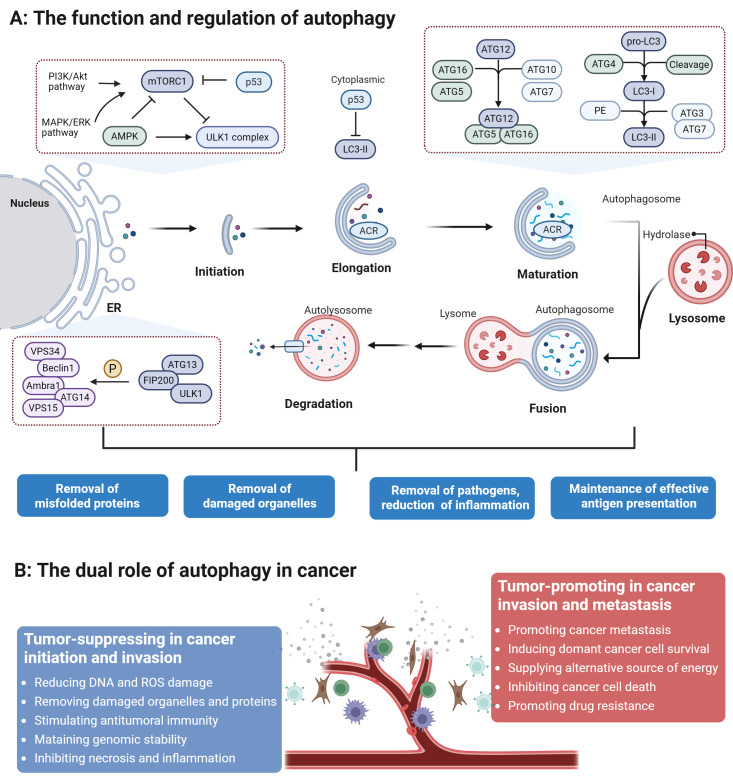
** The functions and regulation of autophagy and its dual role in cancer.** Autophagy is a process composed of initiation, nucleation, elongation and maturation, and fusion and degradation, which regulates tumor proliferation and metastasis by degrading cellular proteins or organelles, thereby playing a dual role in cancer.

In addition to transcriptional control, multiple steps within the autophagy core machinery are critically regulated by posttranscriptional mechanisms, especially noncoding microRNAs (miRNAs) 35. Recent findings reveal that specific miRNAs orchestrate autophagy by directly targeting core autophagy-related (ATG) genes. For example, miR-30a binds to the 3'-UTR of *BECN1* mRNA to suppress phagophore nucleation, while miR-101 limits autophagic capacity by targeting *ATG4D* 36,37. Furthermore, certain miRNAs, such as miR-20a, modulate cellular sensitivity to stress by targeting *ATG16L1* 38. targeting core autophagy-related (ATG) genes and regulatory components, miRNAs orchestrate autophagy across its key stages, from initiation and phagophore formation to autophagosome maturation. These miRNAs often possess a dual regulatory capacity, functioning as either activators or suppressors depending on the cellular context. Their profound impact on development, oncogenesis, and therapeutic resistance highlights their potential as crucial biomarkers and therapeutic targets for modulating autophagy-related pathways (Figure 1).

## 2. Autophagy in the Regulation of Cancer Hallmarks

### 2.1 Proliferative Hallmarks

The core proliferative capabilities of cancer cells form the foundation for malignant transformation. We classify self-sufficiency in growth signals, insensitivity to anti-growth signals, evading apoptosis, and limitless replicative potential as core proliferative hallmarks. This classification is based on the premise that these four features collectively confer on cancer cells the most fundamental survival advantage: the ability to circumvent the growth constraints imposed on normal cells. These hallmarks function synergistically to ensure the unlimited expansion of cancer clones through distinct mechanisms. For example, self-sufficiency in growth signals continuously drives cell cycle progression, insensitivity to anti-growth signals removes critical brakes on cell division, evading apoptosis enables survival under stressful conditions, and limitless replicative potential provides the capacity for infinite propagation essential for long-term tumor evolution. In essence, this group of hallmarks collectively establishes the core capability of cancer cells to persist and proliferate indefinitely.

#### 2.1.1 Mechanisms of Proliferative Hallmarks

The most fundamental hallmark of cancer is the ability of tumor cells to sustain continuous proliferation. This is primarily achieved through aberrant activation of growth factor signaling, constitutive activation of downstream pathways, and dysregulation of cell cycle control mechanisms.

A primary mechanism is autocrine stimulation, in which tumor cells simultaneously produce growth factor ligands such as TGF-α and express the corresponding receptors including EGFR, thereby establishing a self-sustaining signaling loop 39. Alternatively, cancer cells can paracrine stimulation of the stroma, sending signals to normal cells in the tumor-associated stroma, which in turn supply the cancer cells with essential growth factors 40. Receptor signaling is further deregulated by elevating receptor levels on the cell surface, making cells hyper-responsive to otherwise limiting ligand concentrations, or through structural alterations in receptors that facilitate ligand-independent, constitutive activation. Receptor tyrosine kinases (RTKs), such as EGFR, HER2, and FGFR, are frequently overexpressed in various cancers, resulting in persistent activation of downstream signaling cascades, including MAPK, PI3K/AKT, and JAK/STAT, thereby promoting uncontrolled cell proliferation 41. Growth factor independence is also achieved via constitutive activation of downstream signaling components such as mutant RAS and BRAF, which obviates the need for ligand-receptor interaction. Critically, cancer cells frequently disrupt negative-feedback mechanisms designed to attenuate proliferative signaling. For instance, oncogenic RAS mutations impair its intrinsic GTPase activity, turning a transient signal into a persistent one 42. Similarly, loss of the PTEN phosphatase, which degrades the PI3K product PIP3, leads to constitutive PI3K/AKT pathway activation 43.

Concurrently, insensitivity to antigrowth signals allows cancer cells to bypass physiological checkpoints, primarily through the functional inactivation of the retinoblastoma (Rb) and p53 tumor suppressor networks. The RB protein acts as a master integrator of diverse extracellular and intracellular signals, functioning as a critical gatekeeper that decides whether a cell proceeds through the cell cycle. Its inactivation through hyperphosphorylation by overactive cyclin D-CDK4/6 complexes, loss of CDK inhibitors such as p16INK4a, or direct mutation releases E2F transcription factors, thereby driving unimpeded G1/S phase transition 44. TP53 functions as a critical intracellular sensor that halts proliferation or triggers apoptosis in response to severe DNA damage, nucleotide depletion, or suboptimal oxygenation 45.

Programmed cell death serves as a natural barrier to cancer. Tumor cells evolve strategies to evade apoptosis triggered by various stresses encountered during tumorigenesis or therapy. This is typically achieved by shifting the balance of pro- and anti-apoptotic proteins such as the Bcl-2 family or by disrupting death receptor signaling. Specifically, the intrinsic apoptotic program is thwarted when elevated anti-apoptotic proteins (such as Bcl-2, Bcl-xL, and Mcl-1) bind and suppress pro-apoptotic triggers like Bax and Bak. This sequestration prevents Bax and Bak from disrupting the mitochondrial outer membrane, thereby blocking the release of cytochrome c and the subsequent activation of the executioner caspase cascade 46. Tumors further evade apoptosis by inactivating TP53, which eliminates the upstream DNA-damage sensing circuitry that normally induces BH3-only pro-apoptotic proteins like Noxa and Puma 47.

Ultimately, the limitless replicative potential of cancer cells is predominantly achieved by circumventing the natural barriers of replicative senescence and crisis, which limit the proliferation of normal somatic cells to a finite number of divisions. This limitation is governed by the progressive shortening of telomeres accompanied with each cell division. In normal cells, telomere erosion eventually triggers a durable proliferation arrest known as senescence. However, cancer cells tend to maintain telomere length to achieve a state of immortalization, typically via the reactivation of telomerase (TERT) or the alternative lengthening of telomeres (ALT) pathway 48. Cancer cells can reactivate telomerase, a ribonucleoprotein enzyme that adds telomeric repeats to chromosome ends. Others utilize the ALT pathway, a homologous recombination-based mechanism. By counteracting telomere shortening, these mechanisms allow cancer cells to bypass the senescence and crisis barriers hardwired as anticancer defenses.

#### 2.1.2 Autophagy Crosstalk with Proliferative Hallmarks

Rather than functioning as isolated phenomena, the core proliferative hallmarks operate as a highly interconnected signaling network. The starting point of the cancer cell proliferation network is that cancer cells obtain continuous pro-proliferation signal input independent of exogenous signals through the mutation and amplification of growth factor/receptor genes or the establishment of autocrine/paracrine loops. In this process, abnormal proliferation will activate tumor suppressor proteins such as p53, and cancer cells can resist growth signal inhibitory signals by inactivating the RB pathway, the p53 pathway, or degrading cell cycle inhibitors. In addition, abnormal proliferation can also cause energetic and metabolic stress. Subsequently, cancer cells resist suicide programs triggered by metabolic collapse, DNA damage, or tumor suppressor signals by upregulating anti-apoptotic proteins and inhibiting pro-apoptotic proteins. Together, these hallmarks constitute a singular, highly integrated proliferative network. Finally, the activation of the telomere maintenance mechanism provides time conditions for cancer cell proliferation by preventing replicative senescence triggered by telomere shortening.

Autophagy dynamically integrates into the signaling networks of all core proliferative hallmarks. Its activity is precisely tuned by oncogenic signals and tumor microenvironmental stresses, enabling cancer cells to maintain the delicate balance between anabolic biosynthesis and catabolic recycling necessary for relentless proliferation. At the uppermost stage of signaling ignition, autophagy regulates the growth factor/receptor and autocrine/paracrine. First, the loss of autophagy-related genes such as ATG7 or ATG16L1 impairs EGFR endocytosis and recycling, disrupting downstream signaling and cell survival 49. EGFR-overexpressing tumor cells are notably dependent on autophagy for sustained growth and survival 50. Autophagy has also been shown to modulate mutant p53 stability, influencing its accumulation or degradation 51. Besides, autophagy mediates the extracellular secretion of cytokines, such as ATP, an important autocrine/paracrine signaling molecule involved in various cellular functions 52. In drug-resistant melanoma, knockdown of different autophagy genes can inhibit the autophagy-driven extracellular ATP-dependent autocrine-paracrine pathway, thereby attenuating the invasiveness of tumor cells 53. Autophagy also acts as an intermediate molecule in paracrine loops. Paracrine NGF secreted by pancreatic cancer cells can activate autophagy in surrounding Schwann cells, thereby enhancing pancreatic cancer growth and nerve invasion 54. Autophagy also serves as a downstream effector of various growth factors or autocrine/paracrine loops. In the context of hyperactive growth signaling, tumor cells frequently co-opt autophagy to endure the resulting metabolic and oxidative stress. Autophagy degrades intracellular proteins, lipids, and organelles, thereby releasing metabolites such as amino acids and fatty acids that support cell survival under nutrient-limited conditions, contributing to metabolic self-sufficiency. For instance, in RAS-driven tumors, elevated oxidative stress triggers autophagy (specifically mitophagy) to clear damaged mitochondria, thereby sustaining oxidative metabolism and cellular viability 55. In p53-deficient tumors, autophagy compensates for the loss of p53-mediated stress responses, allowing cells to survive and proliferate despite damage or metabolic stress 56. Hypoxia-inducible factor 1α (HIF-1α) not only upregulates pro-angiogenic factors but also transcriptionally activates key autophagy genes like NIX、Beclin 1、ATG5、BNIP3、PIK3C3、ATG7, and ATG9A 57. HIF-1α-induced autophagy has been reported to be associated with the progression of various cancers 58. HIF-1α also induces mitophagy and inhibits mitochondrial biogenesis, thus avoiding cell death. It is also reported that hypoxia-induced NRF2 will change the nutrient acquisition pathway and absorb nutrients outside the cell through macropinocytosis to escape the energy pressure caused by autophagy inhibition 59. Moreover, dysregulated autophagy leads to p62/SQSTM1 accumulation, which activates NRF2 and inhibits mTOR, disturbing growth signal homeostasis 60. Restoring autophagy removes excess p62 and re-establishes the intracellular balance.

Moreover, other types of selective autophagy contribute significantly. Lipophagy (selective degradation of lipid droplets) releases free fatty acids for β-oxidation or membrane synthesis, providing both energy and building blocks 61. Reticulophagy manages endoplasmic reticulum stress, preventing the sustained activation of the unfolded protein response, which can lead to growth arrest 62. Centrosome amplification often occurs in tumors and causes genetic instability to promote tumor progression. However, many cancer cells proliferate despite lacking centrosomes, resulting from TFEB- and TFE3-dependent autophagy activation, which supports cancer proliferation in the absence of centrosomes 63. There is also bidirectional crosstalk between TGF-β signaling and autophagy. In normal cells, TGF-β induces autophagy via Smad2/3, thereby synergistically suppressing proliferation. However, in hepatocellular carcinoma cells, autophagy promotes Smad repression, thereby impairing TGF-β-mediated growth inhibition 64.

As the cascade forcefully progresses to midstream deregulation, tumor cells actively co-opt autophagy to dismantle growth-inhibitory mechanisms and evade cellular senescence. Autophagy can disrupt tumor resistance to anti-growth signals through the degradation of cyclins, thus bypassing cell cycle checkpoints. CMA mediates the autophagic degradation of these specific molecules. Autophagy receptors such as p62/SQSTM1 and NBR1 can bind ubiquitinated p27, targeting it for autophagic degradation 65. This facilitates escape from p27-mediated G1 arrest and promotes deregulation of the Rb pathway, and blocking autophagy can inhibit liver cancer cell proliferation. In hepatocellular carcinoma, autophagy can also degrade Cyclin D1, thereby maintaining cell cycle progression in the absence of growth-suppressive signals 66. Autophagy inhibition can also synergize with CDK4/6 inhibitors to suppress proliferation and induce senescence in breast cancer cells 67. Impaired autophagy is consistently associated with the hallmarks of senescent cells. Mitophagy maintains proliferative capacity by eliminating damaged mitochondria and reducing ROS and DNA damage, thereby mitigating senescence-induced and DDR-based p53/p16-mediated growth suppression 68. However, autophagy has also been shown to suppress aberrant tumor proliferation by mediating the degradation of CDK2 69.

When facing the severe stress generated by this forced division, the network relies on autophagy for downstream acute protection against intrinsic and extrinsic death cascades. One key antiapoptotic mechanism is mitophagy, the selective autophagic removal of damaged mitochondria. By clearing these organelles, autophagy reduces mitochondrial ROS production and prevents the activation of apoptosis 70. PINK1-Parkin-mediated mitophagy has been demonstrated to regulate apoptosis by modulating the ratio of Bcl-2 and either BAX or BAK 71. Autophagy also stabilizes mitochondrial membrane integrity and suppresses BAX/BAK-mediated pore formation, limiting MOMP. Additionally, autophagy can degrade proapoptotic effectors such as caspase-8, thereby attenuating apoptosis 72. Crucially, the autophagic machinery can directly intercept and neutralize the physical components of the apoptotic cascade. Selective macroautophagy driven by receptors like p62/SQSTM1 has been shown to degrade caspases, particularly Caspase-8 and Caspase-9 73,74. CMA can mediate the levels of autophagic degradation of mutant P53, and inhibiting autophagy can increase the death sensitivity of tumor cells 75.

Recent studies also indicate that endoplasmic reticulum autophagy promotes tumorigenesis by inhibiting tumor cell apoptosis 62. The autophagy protein RUBCNL is also involved in inhibiting RIPK1 kinase-dependent apoptosis 76. Conversely, autophagy may also promote apoptosis under certain conditions. When the apoptotic machinery is defective, excessive autophagy can lead to autophagic cell death, a nonclassical death modality characterized by excessive self-digestion. Key mediators of this interplay include PINK1, Beclin1, and others 77. Caspase cleavage of Beclin1 enhances the proapoptotic activity of Bcl-2, promoting cytochrome c release and amplifying apoptosis 78. Furthermore, Beclin1 negatively regulates the antiapoptotic protein Mcl-1; depletion of either protein can stabilize the other, reflecting a reciprocal feedback mechanism 79.

Finally, for the signaling network to achieve terminal consolidation, telomere maintenance strategies are intimately intertwined with autophagic regulation. Mitophagy reduces mitochondrial ROS, which are known to preferentially damage telomeric DNA, thereby slowing telomere erosion and senescence 70. Autophagy also regulates telomerase activity. TERT can bind to and suppress mTORC1, triggering autophagy, which, in turn, stabilizes telomerase localization and activity 80. Additionally, autophagy suppresses DNA damage response signaling, supporting p53-mediated proliferation in the context of sublethal telomeric stress 81.

During a replicative crisis, when telomeres become critically short, cells experience profound genomic instability. In this context, autophagy-dependent cell death has been identified as a key mechanism for eliminating genomically unstable cells. The inhibition of autophagy allows these cells to escape crisis and accumulate chromosomal abnormalities, thereby increasing tumorigenic risk 82. Critically, selective autophagy directly participates in DNA damage response to preserve genomic integrity. For instance, the autophagy receptor TEX264 mediates the lysosomal clearance of topoisomerase 1-DNA crosslinks, promoting repair and survival after genotoxic stress 83. Similarly, the DNA repair protein MLH1 can facilitate nucleophagy, antagonizing 5-FU-induced cytotoxicity 84.

In summary, autophagy serves as an indispensable central orchestrator of cancer's proliferative process. It acts as dynamic response system that integrates inputs from oncogenic signals and microenvironmental stress. Through its broad macroautophagy function and specialized forms like mitophagy, lipophagy, and CMA, autophagy comprehensively fuels the metabolic engine of autonomous growth, actively disables growth suppressors, provides a critical barrier against apoptotic death, and supports the mechanisms of immortality.

### 2.2 Dissemination Hallmarks

Angiogenesis and the activation of invasion and metastasis synergistically facilitate both local tumor progression and distant dissemination. In 1971, Folkman first proposed that angiogenesis is essential for solid tumors to grow beyond a volume of approximately 1–2 mm³ 85. When a tumor grows beyond the size that can be supported by oxygen diffusion, the resulting lack of oxygen and nutrients in its core triggers the "angiogenic switch." This process prompts tumor cells to release factors that stimulate the growth of new blood vessels from the surrounding host tissue 86. The establishment of a neovasculature not only restores oxygen and nutrient delivery and eliminates metabolic waste but, more importantly, provides a direct conduit for tumor cell intravasation, thereby establishing the structural prerequisite for subsequent metastatic dissemination 87. Concurrently, tumor cells undergo epithelial-mesenchymal transition, leading to the loss of cell-cell adhesion and acquisition of migratory capabilities. Through the secretion of matrix metalloproteinases, they degrade components of the extracellular matrix, thereby facilitating breaching of the basement membrane, a critical physical barrier 88. This enables their intravasation into blood or lymphatic vessels, followed by circulation to distant organs and eventual colonization within permissive microenvironments to form metastatic lesions. Collectively, these two hallmarks constitute the fundamental framework driving the transition of cancer from a localized pathology to a systemic disease.

#### 2.2.1 Mechanisms of Dissemination Hallmarks

When a tumor grows beyond the size supported by simple oxygen diffusion, the resulting severe hypoxia and nutrient deprivation trigger a critical "angiogenic switch," which is governed by a precarious balance between countervailing inducers and intrinsic inhibitors. The most pivotal pro-angiogenic inducer is VEGFA, whose expression is upregulated by both hypoxia (via HIF-1α stabilization) and oncogenic signaling. VEGF-A signals through receptor tyrosine kinases on endothelial cells to promote their survival, proliferation, and migration 89. Conversely, thrombospondin-1 is a key endogenous inhibitor of angiogenesis that counteracts these pro-angiogenic stimuli 90. Beyond VEGF transcription**,** the metastatic cascade is a highly inefficient and energetically demanding process, fundamentally driven by epithelial-mesenchymal transition (EMT) and extensive extracellular matrix (ECM) remodeling. Orchestrated by transcription factors (Snail, Twist, ZEB1/2), EMT leads to loss of E-cadherin, acquisition of a motile, mesenchymal phenotype, and enhanced resistance to apoptosis. Cells then degrade the extracellular matrix and basement membrane via MMPs to invade locally, intravasate into circulation, survive anoikis, extravasate at distant sites, and finally colonize to form metastases 91.

These two hallmarks are functionally intertwined, shared common microenvironmental drivers like hypoxia, simultaneously inducing VEGF-driven angiogenesis and EMT-driven invasion via HIF-1α. Subsequently, the neo-vasculature provides the conduit for intravasation, and invasion fuels angiogenesis through sustains VEGF expression and the angiogenic switch.

#### 2.2.2 Autophagy Crosstalk with Dissemination Hallmarks

Rather than acting on angiogenesis and metastasis in isolation, the autophagic machinery operates as a master spatial and temporal coordinator throughout the entire dissemination cascade. From the initial hypoxic insult to terminal distant colonization, autophagy dynamically dictates the fate of tumor cells, endothelial cells, and the surrounding stroma to ensure successful systemic spread 92.

At the genesis of the dissemination cascade, under hypoxia and nutrient stress, autophagy is activated via the HIF-1α- and AMP-activated protein kinase (AMPK)–mTOR pathways, enabling tumor cell adaptation by removing damaged mitochondria, reducing ROS, stabilizing HIF-1α, and thereby increasing VEGF expression 93. Simultaneously, autophagy machinery directly regulates the intercellular communication required for both angiogenesis and pre-metastatic niche formation. Tumor cell autophagy also promotes the secretion of proangiogenic exosomes; for example, CEP55-driven exosome release enhances angiogenesis in non–small-cell lung cancer via mTOR activation 94. Mechanistically, autophagosomes fuse with multivesicular bodies to form “amphisomes,” which traffic to the plasma membrane to release exosomes 95. ATG5 has been implicated in regulating extracellular vesicle secretion via lysosomal pathways 96.

Within the compromised tumor vasculature, autophagy acts as a critical quality control mechanism. Basal autophagy recycles macromolecules and clears damaged organelles or protein aggregates, preserving endothelial cell integrity and function 97. Loss of endothelial autophagy induces IL-6-dependent endothelial-to-mesenchymal transition and fibrosis 98. Conversely, in early tumor stages of non-small cell lung cancer cells, autophagy activation may degrade proangiogenic components such as VEGF or endothelial survival factors, exerting antiangiogenic effects 99. Furthermore, autophagy acts as a potent mediator of therapeutic resistance during vascular targeting. Excessive autophagy in endothelial cells can also trigger cell death, further inhibiting vessel growth. Furthermore, regarding resistance associated with anti-VEGF therapies that inhibit tumor angiogenesis, studies have demonstrated that this resistance correlates with increased transcription of the autophagy enhancer protein RUBCNL, which is mediated by histone acetylation and ultimately interacts with Beclin1 to promote autophagy 100.

As tumor cells initiate metastasis process, autophagy physically dismantles epithelial barriers and drives ECM degradation. It contributes to invasion and metastasis by facilitating EMT and ECM remodeling. For example, p62/SQSTM1-mediated autophagic degradation of E-cadherin disrupts cell–cell adhesion and promotes EMT 101. Autophagy also enhances the expression and secretion of MMPs, accelerating matrix degradation 102. Additionally, autophagy regulates exosome biogenesis and secretion, contributing to the formation of premetastatic niches. EMT can confer cancer stem cell-like properties that promote metastasis, recurrence, and therapeutic resistance. Autophagy inhibition via ATG7 or BECN1 knockdown reduces IL-6 secretion and downstream STAT3-mediated propagation of cancer stem cells, thereby limiting their metastatic potential 103. ATG7 deficiency can also drive the transformation of fibroblasts into CAFs, which alter the tumor microenvironment via exosomes. This process mediates SCARB1 gene suppression in breast cancer cells, thereby promoting cancer cell metastasis and driving disease progression 104. Finally, cancer stem cells (CSCs), which possess self-renewal and long-term proliferative capacity, rely on elevated autophagic activity to sustain a low-ROS environment and maintain their replicative fitness under metabolic stress, thereby reinforcing the "limitless replicative potential" phenotype. For instance, inhibiting autophagy (Beclin1 knockdown) will lead to an increase in ROS, thereby destroying the stemness characteristics and tumorigenic ability of breast cancer stem cells 105. Autophagy also interacts with multiple core signaling pathways that maintain the stemness of CSCs, like stabilizing HIF-1α and regulating the activity of β-catenin, thereby affecting the stemness of CSCs 106.

In summary, autophagy acts as a critical regulatory node integrating both processes. Under the stress conditions that characterize this integrated network, autophagy supports angiogenesis by maintaining endothelial cell health and providing metabolic precursors for VEGF production. It facilitates invasion-metastasis by modulating the secretion of pro-invasive factors and regulating tumor cell migratory capacity by influencing the degradation of key epithelial-mesenchymal transition transcription factors, including Snail and Twist 107.

### 2.3 Stress and Plasticity Hallmarks

Here, we categorize genomic instability and mutation, metabolic reprogramming, non-mutational epigenetic reprogramming, and unlocking phenotypic plasticity together as "stress and phenotypic remodeling" hallmarks. This classification is based on the premise that these four features collectively equip cancer cells with the capacity to adapt and survive under environmental stress from distinct yet complementary mechanistic dimensions. Genomic instability and mutation serve as the genetic foundation for tumor adaptation. Under persistent stress conditions such as hypoxia, nutrient deprivation, and therapeutic exposure, defects in DNA repair systems lead to a markedly elevated mutation rate 108. This instability generates substantial genetic diversity within the tumor cell population, enabling the selection and survival of rare clones harboring advantageous mutations. By doing so, it fuels tumor evolution and provides the essential raw material for subsequent adaptive changes. Metabolic reprogramming represents a strategic adaptation to energetic and biosynthetic demands. Even in the presence of adequate oxygen, tumor cells preferentially engage in aerobic glycolysis, which not only supports rapid proliferation by supplying essential biosynthetic precursors but also sustains cellular functions under hypoxic or nutrient-limited conditions 109. This metabolic flexibility enables tumor cells to maintain viability and functionality despite fluctuating microenvironmental resources. Non-mutational epigenetic reprogramming offers a rapid and reversible mechanism for stress adaptation. In response to hypoxia, inflammation, or therapeutic pressure, tumor cells can swiftly alter gene expression patterns through DNA methylation and histone modifications, circumventing the need for uncertain and time-consuming genetic mutations 110. This dynamic regulatory capacity allows tumors to mount immediate adaptive responses to environmental fluctuations, thereby enhancing their resilience. Unlocking phenotypic plasticity represents the functional culmination of the aforementioned mechanisms. Through the integrated effects of genetic variation, metabolic adaptation, and epigenetic modulation, tumor cells acquire the ability to transition between distinct cellular states, such as shifting from a proliferative to an invasive phenotype or entering a slow-cycling, drug-tolerant state 111. This state-switching capability enables tumor cells to dynamically adjust their behavior in response to changing environmental conditions, thereby preserving their survival advantage throughout disease progression.

#### 2.3.1 Mechanisms of Stress and Phenotypic Plasticity Hallmarks

During tumor progression, cancer cells face a range of intrinsic and extrinsic stresses, including hypoxia, oxidative stress, energy depletion, and endoplasmic reticulum (ER) stress. These stresses are interwoven and collectively form a harsh yet selective microenvironment that drives tumor adaptation. The characteristics acquired by tumors in response to these stresses ultimately constitute the hallmarks of cancer. For instance, intratumoral hypoxia is a defining feature of heterogeneous solid tumors. Tumor hypoxia has been intensively studied as an environmental factor that promotes genomic instability in cancer cells 112. Initial studies using reporter gene assays or DNA break assays in cell lines concluded that cells under hypoxic conditions activate DNA fragile sites and undergo gene amplification, microsatellite instability (MSI), and base-pair mutations 108. Furthermore, endogenous factors include reactive oxygen species (ROS)-induced DNA oxidative damage, in which ROS attack DNA, leading to base modifications and strand breaks. Exogenous factors such as ultraviolet and ionizing radiation, as well as chemical mutagens, can directly cause DNA damage. Tumor cells often exhibit deficiencies in DNA damage response (DDR) mechanisms, including dysregulation of ATM/ATR signaling pathways, impaired Chk1/Chk2 function, and defective repair pathways such as base excision repair (BER), nucleotide excision repair (NER), and homologous recombination 113.

Metabolic reprogramming is a hallmark that enables tumor cells to meet acute energy and biosynthetic demands, resist stress, and maintain redox balance. Major mechanisms include the Warburg effect, glutaminolysis, fatty acid metabolism, and de novo lipogenesis. Metabolic reprogramming also represents a stress response of tumors to external pressures. For instance, hypoxia in cancer cells leads to reduced mitochondrial activity and decreased ATP production, prompting a metabolic switch to glycolysis as a means of generating energy independently of oxygen 114. Furthermore, the aberrant vasculature within the tumor microenvironment creates local deficiencies in amino acids, fatty acids, and other nutrients, thereby forcing tumor cells to upregulate glutamine metabolism and fatty acid metabolism 115. These shifts in cancer metabolism were regulated by several stress-responsive factors, including the mammalian target of rapamycin complex 1 (mTORC1), the myelocytomatosis viral oncogene homolog (c-Myc), hypoxia-inducible factor-1α (HIF-1α), activating transcription factor 4 (ATF4), nuclear factor erythroid 2–related factor 2 (NRF2), and sterol regulatory element–binding protein 1 (SREBP1) 116.

Non-mutational epigenetic reprogramming refers to a process by which cancer cells reset their gene expression programs through epigenetic modifications in the absence of genetic mutations, thereby acquiring malignant phenotypes or adapting to environmental changes. For instance, hypoxia induces HIF-α-dependent epigenetic susceptibility in triple-negative breast cancer, leading to impaired immune effector function and resistance to anti-PD-1 immunotherapy 117. Moreover, hypoxia can also induce rapid, HIF-independent histone methylation alterations. Under hypoxic conditions, the Jumonji C (JMJC) domain-containing lysine demethylases KDM5A and KDM6A are inhibited, resulting in hypermethylation of histone marks associated with both gene activation and repression 118.

Phenotypic plasticity is an emerging hallmark of cancer that promotes tumor heterogeneity, progression, and therapy resistance 119. Phenotypic plasticity enables cancer cells to dynamically alter their differentiation state and acquire stem cell-like properties, thereby increasing their adaptability and survival. Cancer cells gain stemness through metabolic reprogramming. Studies indicate that pancreatic cancer stem cells (PaCSCs) exhibit distinct metabolic features, including enhanced oxidative phosphorylation (OXPHOS) and increased mitochondrial function 120. The acquisition of stemness also depends on the redirection of tricarboxylic acid (TCA) cycle intermediates into lipogenic pathways. ACC1-mediated de novo fatty acid synthesis promotes acetyl-CoA consumption, leading to increased mitochondrial fission and subsequent induction of stemness via acetylation of FIS1 121.

#### 2.3.2 Autophagy Crosstalk with Stress and Phenotypic Plasticity Hallmarks

During tumor progression, cancer cells are constantly exposed to a variety of microenvironmental stresses, including hypoxia, oxidative stress, nutrient deprivation, and endoplasmic reticulum (ER) stress. To survive and thrive under these adverse conditions, tumors evolve a set of adaptive features, including genomic instability and mutation, metabolic reprogramming, non-mutational epigenetic reprogramming, and phenotypic plasticity, which together constitute the stress and plasticity hallmarks. Autophagy, a highly conserved homeostatic mechanism that responds to diverse cellular stresses, serves as a central hub connecting these hallmarks. By sensing and integrating multiple stress signals, autophagy concurrently regulates several stress-responsive pathways, thereby establishing extensive crosstalk with each of the above hallmarks.

Autophagy crosstalks with these four stress-driven hallmarks primarily through shared molecular nodes, among which reactive oxygen species (ROS) serve as a key integrator. Autophagy buffers ROS levels by selectively removing damaged mitochondria (mitophagy) and reducing metabolic stress. When autophagy is functional, ROS are maintained at a low-to-moderate range that limits DNA damage and chromosomal aberrations, thereby suppressing genomic instability. Conversely, impaired autophagy leads to ROS accumulation, which promotes double-strand breaks and mutagenesis, linking autophagic flux to mutation rates. For instance, YM155, a targeted inhibitor of BIRC5, has been shown to suppress autophagy, leading to elevated reactive oxygen species (ROS) levels, impairing DNA damage repair, and ultimately resulting in genome instability 122. Importantly, ROS also act as signaling molecules that reprogram cellular metabolism. Under hypoxic conditions in uveal melanoma (UM), BNIP3-dependent mitophagy alleviates mitochondrial dysfunction, boosts OXPHOS, and concurrently lowers mtROS levels. This cascade impairs HIF-1α stability and thereby inhibits glycolysis 123. These metabolic changes, in turn, affect the availability of epigenetic cofactors such as α-ketoglutarate and lactate, thereby modulating DNA and histone methylation/lactylation. In lenvatinib-resistant HCC, increased glycolysis induces lactate-mediated lysine lactylation of IGF2BP3, which stabilizes PCK2 and NRF2 mRNAs to reprogram serine metabolism and boost antioxidant defenses. This metabolic shift elevates S-adenosylmethionine (SAM), promoting m6A methylation of PCK2 and NRF2 mRNAs, thereby sustaining antioxidant capacity and driving lenvatinib resistance 124. Mitophagy maintains tricarboxylic acid cycle function by clearing damaged mitochondria, ensuring a sustained supply of metabolites, including α-ketoglutarate (α-KG). As an essential cofactor for TET dioxygenases and JMJC domain histone demethylases, α-KG directly regulates DNA and histone demethylation. Autophagy deficiency disrupts the α-KG/succinate ratio, leading to abnormal H3K27me3 accumulation and gene silencing. Furthermore, autophagy provides acetyl-CoA through lipolysis, a critical substrate for histone acetylation 125. Autophagy plays a critical role in regulating phenotypic plasticity and influences cancer progression and therapeutic response through multiple mechanisms. In pancreatic cancer stem cells, increased ISG15 expression and ISGylation of proteins are essential for sustaining metabolic flexibility. Loss of ISG15 leads to the accumulation of dysfunctional mitochondria, reduced OXPHOS, and impaired mitophagy, ultimately compromising the self-renewal and tumorigenic capacity of PaCSCs 126. LC3A-mediated autophagy cross talks with SOX2 proliferation signaling to regulate mitochondrial metabolism and determines cancer cell plasticity 127. Similarly, in glioma-initiating cells (GICs), autophagy suppresses stemness and tumorigenicity by inhibiting Notch1 signaling, a stemness-promoting pathway 128. Thus, autophagy regulates ROS to create a functional crosstalk that simultaneously links all four hallmarks: genomic instability, metabolic reprogramming, non-mutational epigenetic reprogramming, and phenotypic plasticity.

Beyond ROS, autophagy engages multiple additional pathways to crosstalk with these hallmarks. For example, through the regulation of p62, autophagy influences both tumor metabolism and genomic stability. In particular, autophagy can induce an alternative nutrient acquisition route in pancreatic ductal adenocarcinoma: macropinocytosis (MP), which allows tumor cells to extract extracellular nutrients and utilize them for energy production. The switch from autophagy to MP may be evolutionarily conserved and is not restricted to cancer cells; it depends on activation of the transcription factor NRF2 by the autophagy adaptor p62/SQSTM1 59. p62/SQSTM1 also regulates micronuclear stability, thereby affecting chromosome fragmentation and rearrangements. Mechanistically, the close proximity of micronuclei to mitochondria promotes oxidation-driven homo-oligomerization of p62, which limits ESCRT-dependent micronuclear envelope repair by inducing autophagic degradation 129. Furthermore, hypoxia-inducible factor-1α (HIF-1α) serves as a master regulator that coordinates genomic stability, epigenetic reprogramming, metabolic reprogramming, and differentiation under low-oxygen conditions. On one hand, under hypoxia, autophagy stabilizes HIF-1α, thereby modulating multiple hallmarks 130. On the other hand, HIF-1α activation can reciprocally induce autophagy, which in turn helps cells withstand adverse microenvironmental conditions 58. Together, these nodes establish a multi-layered crosstalk network wherein autophagy simultaneously governs stress adaptation and phenotypic evolution in a context-dependent manner.

### 2.4 Microenvironmental and Immune Hallmarks

The tumor microenvironment and immune regulation involve the intricate interplay of immune evasion, tumor-promoting inflammation, the polymorphic microbiome, and senescent cells. Under normal physiological conditions, immune cells are capable of recognizing and eliminating aberrant cells. However, tumors evade immune attack through multiple mechanisms, including the expression of immune checkpoint molecules and the recruitment of immunosuppressive cells 2. This ability to avoid immune clearance enables tumors to survive and proliferate despite the presence of immune surveillance. Tumor-promoting inflammation facilitates the release of growth factors and cytokines from inflammatory cells, which in turn support tumor cell proliferation, survival, and angiogenesis. The polymorphic microbiome modulates anti-tumor immune responses, with distinct microbial compositions either enhancing or suppressing immunity, and can even influence the efficacy of immunotherapeutic interventions 131. Senescent cells, though growth-arrested, secrete a range of inflammatory factors, growth factors, and matrix-remodeling enzymes. These secretory products reshape the local microenvironment, thereby exerting dual effects. On one hand, they may suppress tumor progression by recruiting immune cells that eliminate malignant cells; on the other hand, they can promote tumor growth by establishing a pro-inflammatory milieu 132. Collectively, these four hallmarks constitute the ecosystem of the tumor microenvironment.

#### 2.4.1 Mechanisms of Microenvironmental and Immune Hallmarks

A key factor enabling tumor proliferation and growth within the host is its ability to remodel the local immune system, establishing an immunosuppressive and chronically inflamed microenvironment. In this setting, immune cells are either inactivated or rendered incapable of recognizing tumor cells, thereby allowing the tumor to evade host immune surveillance. The adaptive strategies tumors employ to resist immune surveillance and destruction ultimately give rise to a set of hallmark capabilities. Cancer cells evade immune destruction through multiple mechanisms, with disruption of the MHC-I antigen presentation pathway being central. Defects in components such as immunoproteasome subunits, TAP, tapasin, ERAP1/2, MHC-I heavy chain, β2-microglobulin, or interferon signaling lead to reduced surface MHC-I expression, allowing tumors to escape CD8⁺ T-cell recognition 133,134. In parallel, tumors upregulate PD-L1, which suppresses the PI3K/AKT and Ras/MAPK/ERK pathways, induces T-cell exhaustion, and is further enhanced by STAT3-mediated PD-L1 expression, thereby strengthening immunosuppression 135.

Tumor-promoting inflammation serves as an enabling hallmark that facilitates malignant progression by establishing a chronic, non-resolving inflammatory microenvironment. It recruits various cytokines to promote tumor proliferation and metastasis. Concurrently, these inflammatory factors and chemokines orchestrate the recruitment of immunosuppressive cell populations, including tumor-associated macrophages (M2 phenotype), myeloid-derived suppressor cells, and regulatory T cells—into the tumor niche 136. Furthermore, the inflammatory microenvironment upregulates immune checkpoint molecules such as PD-L1 on both tumor and immune cells, driving T-cell exhaustion 137.

As one of the emerging hallmarks of cancer, the polymorphic microbiome exerts its core functions by interacting with immune cells and cancer cells within the tumor microenvironment, thereby influencing tumor initiation, progression, and therapeutic response at multiple levels. *Fusobacterium nucleatum* promotes tumor cell proliferation and inflammatory responses by binding E-cadherin via its FadA adhesin, thereby activating the β-catenin signaling pathway. Moreover, this bacterium recruits myeloid-derived suppressor cells (MDSCs), fostering an immunosuppressive microenvironment 138. The microbiome further influences tumor progression by regulating immune responses. Bifidobacterium enhances dendritic cell function and CD8⁺ T-cell responses, thereby improving the efficacy of immune checkpoint inhibitors such as anti-programmed cell death protein 1 (PD-1)/programmed death-ligand 1 (PD-L1) antibodies. In contrast, certain microbes, including *Fusobacterium nucleatum*, promote immune evasion by activating immune checkpoint molecules such as TIGIT, which suppresses the function of natural killer (NK) cells and T cells 139.

Cellular senescence is a stable cell cycle arrest program that plays a complex and dual role in tumor initiation and progression. In the early stages of tumorigenesis, the induction of cellular senescence prevents unlimited proliferation of premalignant cells, thereby suppressing tumor development. Moreover, senescent cells secrete a variety of SASP factors, which recruit immune cells to eliminate transformed cells. Autophagy can suppress SASP factors, thereby inhibiting inflammation and tumorigenesis. However, these senescence-associated secretory phenotype (SASP) factors also contain numerous pro-inflammatory cytokines that contribute to the formation of an inflammatory microenvironment, thereby promoting tumor growth, metastasis, and immunosuppression. In certain contexts, senescent cells may also exploit autophagy to resist senescence and survive, thereby further promoting SASP secretion and facilitating tumorigenesis 140.

#### 2.4.2 Autophagy Crosstalk with Microenvironmental and Immune Hallmarks

Chronic inflammation serves as a common thread linking immune evasion, tumor-promoting inflammation, the polymorphic microbiome, and senescent cells. Autophagy, as a master regulator of inflammation, concurrently influences all four hallmarks by controlling the initiation, amplitude, and resolution of inflammatory responses in the tumor microenvironment.

Autophagy can suppress the tumor-promoting inflammatory microenvironment through multiple pathways. First, autophagy is closely associated with the activation of the NLRP3 inflammasome and the subsequent release of IL-1β. In breast cancer, knockdown of ULK1 impairs mitophagy under hypoxic conditions, resulting in the accumulation of damaged mitochondria and increased production of reactive oxygen species (ROS), which subsequently activate the NLRP3 inflammasome. This aberrant activation disrupts the secretion of soluble cytokines, promotes osteoclast differentiation and maturation, and ultimately facilitates bone metastasis 141. Furthermore, these proinflammatory cytokines also contribute to the establishment of an immunosuppressive microenvironment by recruiting myeloid-derived suppressor cells (MDSCs) and regulatory T cells (Tregs) 142. Importantly, deficiency in the mitophagy-related proteins Parkin and PINK1 results in increased NLRP3 inflammasome activation in response to various NLRP3 agonists 143. In contrast, induction of autophagy significantly reduces the cleavage of pro-caspase-1 and pro-IL-1β, indicating diminished inflammasome activation 144. Importantly, this mechanism also applies to the regulation of the senescence-associated secretory phenotype (SASP) by autophagy in senescent cells. The SASP primarily includes pro-inflammatory factors such as IL-1β, IL-6, and other inflammatory mediators. Studies have reported that impaired autophagy leads to upregulation of the SASP, whereas restoration of autophagy reverses the senescent phenotype by suppressing GATA4, a transcription factor that regulates both senescence and the SASP 145. Consistently, GATA4 is stabilized in senescent cells. Under normal conditions, GATA4 is degraded via p62-mediated selective autophagy; however, this regulatory process is suppressed during senescence, leading to GATA4 stabilization. Stabilized GATA4 in turn activates the transcription factor NF-κB, which initiates the SASP and promotes cellular senescence 146. Moreover, autophagy-dependent glutamine metabolism is crucial for maintaining the SASP; inhibition of autophagy significantly reduces glutaminase activity in senescent cells and impairs the secretion of inflammatory factors 147. Importantly, the role of autophagy in regulating senescence is context-dependent. In early-stage tumors, autophagy acts as a tumor suppressor by maintaining senescence; in advanced cancers, it may support the survival of senescent cells, facilitating tumor recurrence and metastasis 148.

Autophagy couples inflammation to immune evasion via the p62/NF-κB axis. Studies have reported that in macrophages, excessive activation of NF-κB leads to the release of mitochondrial DNA (mtDNA) and mitochondrial reactive oxygen species (mtROS). The damaged mitochondria subsequently undergo Parkin-dependent ubiquitination and are specifically recognized by p62, thereby triggering mitophagic clearance. This process alleviates mitochondrial damage and excessive IL-1β-dependent inflammation, while also preventing macrophage death 149. p62 can also inhibit NF-κB activation by promoting the autophagic degradation of TRAF6, thereby evading host innate immunity 150. Moreover, p62 affects PD-L1 expression and stability in a context-dependent manner. In gastric cancer, autophagy regulates PD-L1 levels via the P62/SQSTM1–NF-κB signaling pathway 151. Therefore, the same autophagic defect that fuels pro-tumor inflammation also facilitates immune evasion, effectively linking these two cancer hallmarks through a shared molecular mechanism. Notably, this interplay is context-dependent. In tumors characterized by high basal NF-κB activity, such as those driven by mutant p53 or oncogenic KRAS, restoring autophagic flux may serve a dual function: simultaneously attenuating inflammation and downregulating PD-L1 expression, thereby potentially enhancing tumor sensitivity to immunotherapy.

Autophagy also connects the microbiome to inflammation and immune evasion. LC3-associated phagocytosis (LAP) is a non-canonical autophagy pathway that uses core autophagy machinery to facilitate phagosome maturation and clearance of engulfed cargo, including apoptotic cells and microbes. Accumulating evidence indicates that autophagic deficiency leads to reduced microbial degradation, thereby promoting the onset of inflammation 152. In one study, glucose-starved macrophages isolated from T316A knock-in mice exhibited a 50% reduction in Atg16L1 protein levels, resulting in impaired autophagic clearance of bacteria and increased expression of the pro-inflammatory cytokine IL-1β. This defective stress-induced autophagy and xenophagy consequently establish a chronic inflammatory state 153. Furthermore, certain microbial metabolites can modulate immune evasion through autophagy. For instance, spermidine derived from probiotics promotes IFN-γ⁺CD4⁺ T cell immunity via autophagy, thereby facilitating hepatitis B virus (HBV) clearance 154. The microbial metabolite of quercetin, 3,4-dihydroxyphenylacetic acid (DOPAC), enhances the expression of BCL2-interacting protein 3 (BNIP3), which in turn promotes mitophagy and improves mitochondrial function, ultimately ameliorating CD8⁺ T cell fitness within the tumor microenvironment 155. Additionally, Fusobacterium nucleatum activates autophagic pathways by targeting the TLR4/MYD88 innate immune signaling axis and specific microRNAs, thereby altering the response of colorectal cancer to chemotherapy 156.

**Figure 2:**
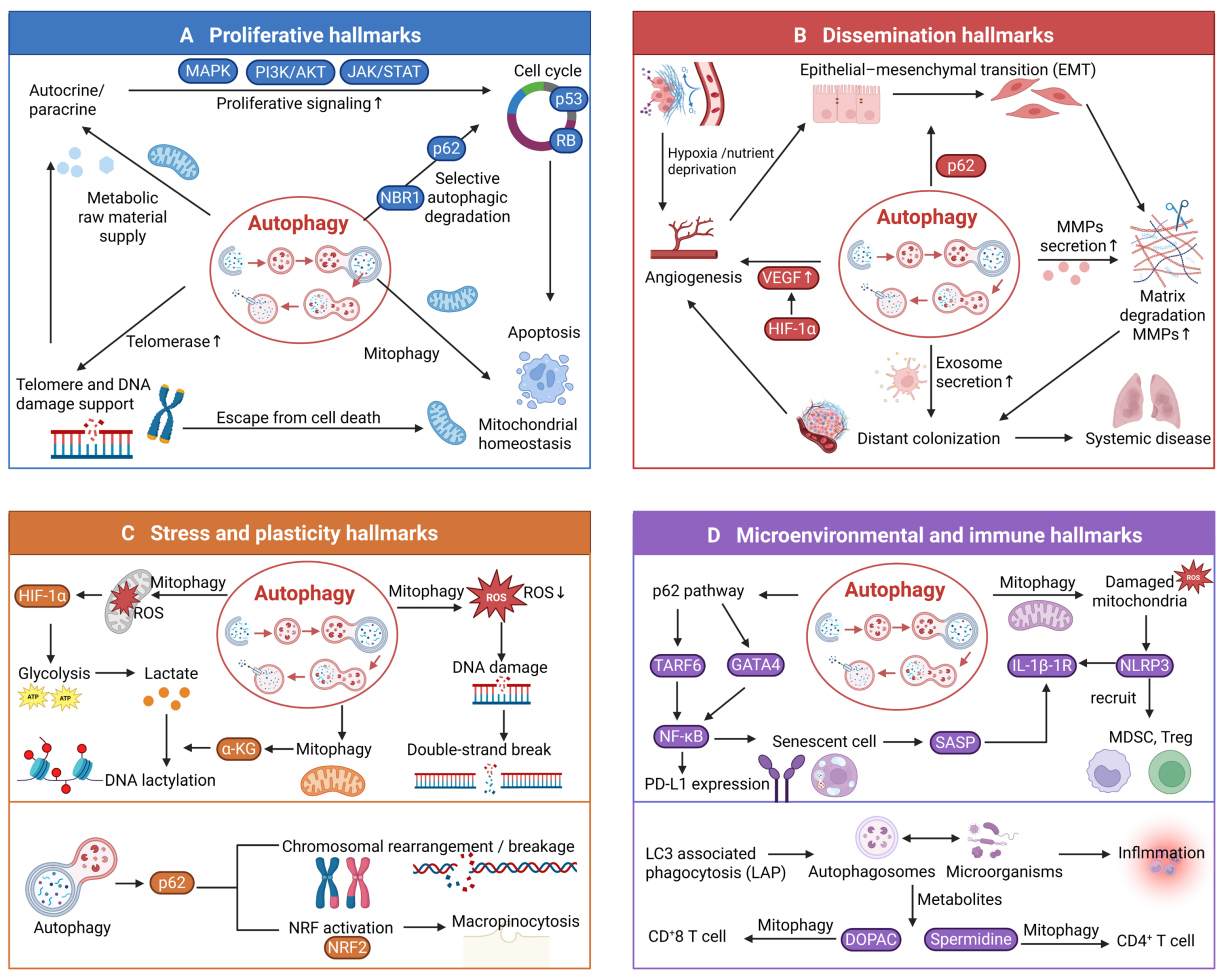
** Molecular mechanisms of autophagy in regulating each hallmark category.** The four quadrants illustrate the core molecular mechanisms by which autophagy regulates each of the four proposed hallmark categories: proliferative hallmarks, dissemination hallmarks, stress and plasticity hallmarks, and microenvironmental and immune hallmarks. As shown, autophagy modulates each category through multiple distinct pathways.

Collectively, these mechanisms illustrate that autophagy positions itself at the center of the inflammation-immunity network. By tuning inflammasome activity, NF-κB signaling, SASP secretion, and LAP-dependent clearance, autophagy simultaneously determines the inflammatory tone of the tumor microenvironment, the efficacy of anti-tumor immunity, the impact of microbial dysbiosis, and the deleterious effects of senescent cell accumulation. This integrated view suggests that strategies aimed at restoring or modulating autophagic flux may have broad therapeutic potential by targeting multiple microenvironmental and immune hallmarks at once (Figure 2).

### 2.5 Integrative Crosstalk: Autophagy as a Central Hub across Hallmark Categories

The preceding sections have described how autophagy engages in crosstalk with hallmarks within each of the four categories. However, autophagy functions as a central hub not by acting on each hallmark in isolation, but by driving core cellular processes that inherently cascade into multiple hallmark categories. For instance, Autophagy-driven metabolic reprogramming generates a cascade of effects across multiple hallmark categories. By degrading macromolecules, autophagy supplies amino acids and nucleotides that fuel biosynthetic pathways, sustaining tumor proliferation. Autophagy also supports the production of α-ketoglutarate (α-KG) , which serve as substrates for TET-mediated DNA demethylation and histone acetylation, respectively, driving non-mutational epigenetic reprogramming 157,158. Furthermore, autophagy-regulated ketone bodies reshape the tumor immune microenvironment by polarizing macrophages toward an immunosuppressive M2 phenotype and driving cytotoxic T cell function 159,160.

Autophagy preserves mitochondrial homeostasis through mitophagy, the selective clearance of damaged mitochondria. This process broadly impacts multiple cancer hallmarks. First, by removing mitochondria that have undergone permeability transition, mitophagy prevents the release of cytochrome c and other pro-apoptotic factors, thereby enhancing apoptosis resistance-a hallmark of sustained proliferation 161. Second, mitophagy limits the leakage of mitochondrial DNA into the cytosol, which otherwise activates the cGAS-STING pathway and triggers type I interferon responses 162. Defective mitophagy leads to chronic cGAS-STING activation, fostering both pro-tumor inflammation and immunosuppression, two key immune hallmarks 163. Third, mitophagy refines cellular metabolism by eliminating dysfunctional mitochondria that produce excessive reactive oxygen species (ROS) and inefficient ATP. This metabolic optimization supports oxidative phosphorylation efficiency and enables the metabolic flexibility required for epithelial–mesenchymal transition and cell migration, which are associated with dissemination hallmarks 164. Fourth, mitophagy-mediated control of ROS levels directly influences the stability of transcription factors such as HIF-1α and NRF2, which regulate angiogenesis, metabolic reprogramming, and antioxidant responses, thereby affecting stress adaptation and plasticity hallmarks 165.

Autophagy facilitates immune evasion through the downregulation of MHC-I and upregulation of PD-L1 via the p62/NF-κB axis. This same NF-κB activation also upregulates matrix metalloproteinases (MMPs) and Snail, thereby directly promoting epithelial–mesenchymal transition (EMT) and invasion, a key dissemination hallmark 166. Sustaining PD-L1 expression and NF-κB signaling requires high metabolic activity; autophagy supports this demand by providing metabolic substrates and maintaining redox balance under oxidative stress, contributing to stress hallmarks such as metabolic reprogramming. Moreover, successful immune evasion enables tumor cells to escape immune-mediated killing, thereby sustaining their proliferative capacity. Concurrently, inflammatory signals—including IL-6 and TNF-α—derived from the immunosuppressive microenvironment activate NF-κB and STAT3, further driving cell cycle progression and reinforcing the proliferative hallmark.

Beyond macroautophagy, other forms of autophagy, such as microautophagy, LC3-associated phagocytosis (LAP), and chaperone-mediated autophagy (CMA), also regulate multiple cancer hallmarks. RNautophagy, a selective type of microautophagy, mediates the transport of cellular RNA into lysosomes for degradation 167. Studies have reported that knockdown of SIDT2, the receptor for RNautophagy, confers enhanced resistance in mice to lung adenocarcinoma and colorectal cancer driven by the oncogenic mutations KRAS^G12D^ and APC^min/+^, respectively. In the intestine, APC^min/+^ Sidt2^-/-^ mice shows the accumulation of double-stranded RNA (dsRNA) is associated with increased phosphorylation of eIF2α and JNK, as well as elevated rates of apoptosis 168. Furthermore, when macroautophagy is inhibited, SNX9 mediates the trafficking of mitochondrial-derived vesicles to lysosomes, thereby inducing mitophagy via microautophagy. This process ultimately compensates for the loss of macroautophagy to maintain mitochondrial fitness 169. For chaperone-mediated autophagy, the study has uncovered a CMA-dependent, proteasome-independent Snail degradation pathway that limits Snail levels in luminal-type breast cancer cells. In TNBC cells, nuclear localization evades this degradation, which drives EMT and metastasis 107. The study has reported that ROS-triggered o8G modification reduces circPLCE1 stability through the RNA-binding protein AUF1. Mechanistically, circPLCE1 inhibits cancer progression by binding to HSC70, increasing its ubiquitination, and thereby modulating ATG5-mediated macroautophagy via the CMA pathway 170. LC3-associated phagocytosis (LAP) is characterized by the formation of single-membrane vesicles decorated with the autophagy protein LC3, which is triggered upon receptor-mediated phagocytosis. In myeloid cells, loss of LC3-associated phagocytosis (LAP) reprograms tumor-associated macrophages (TAMs) toward a pro-inflammatory state. This alteration, triggered by the phagocytosis of dying tumor cells, activates the STING-type I interferon axis, ultimately leading to suppressed tumor growth 171.

**Figure 3:**
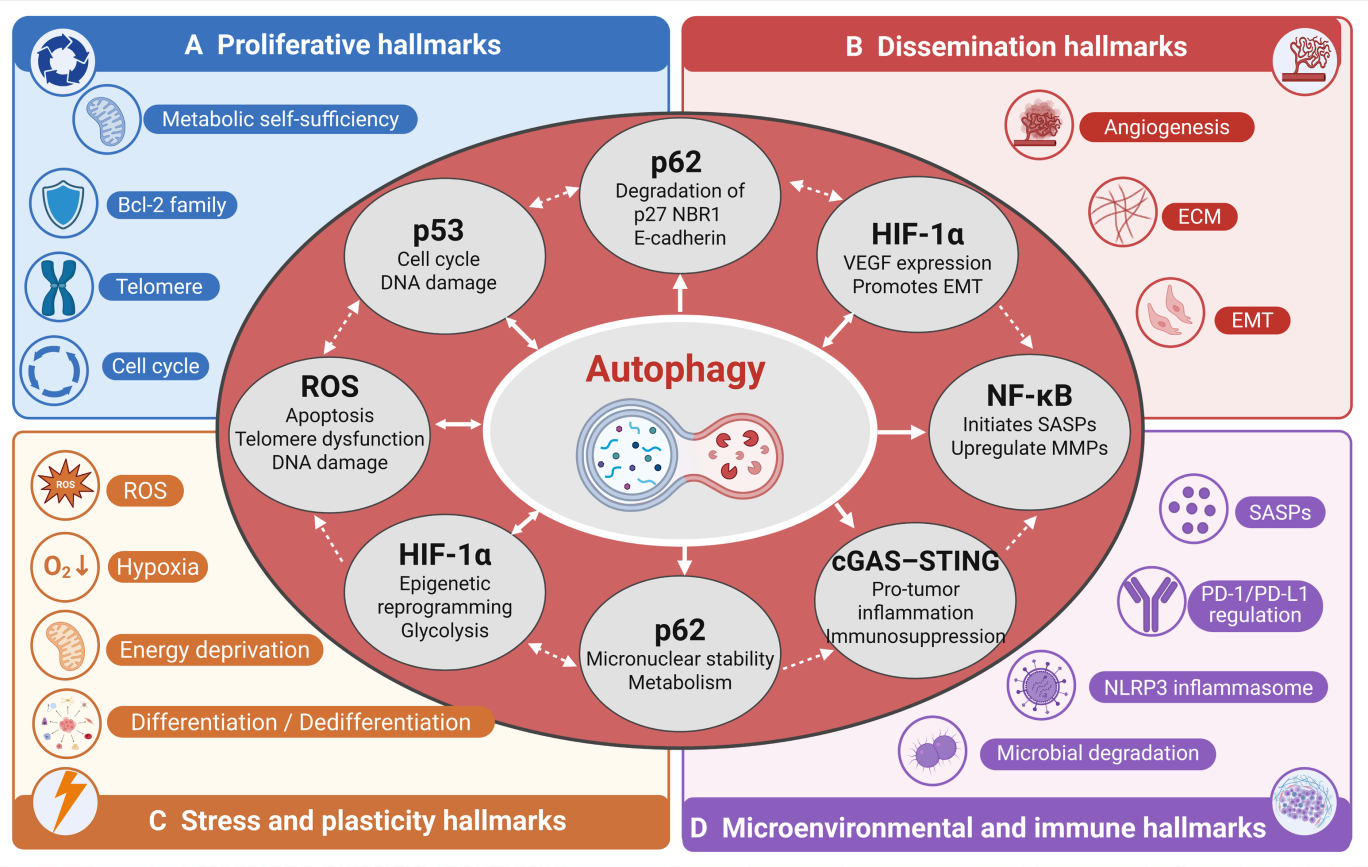
** Key molecular nodes mediating autophagy crosstalk across hallmark categories.** This figure illustrates how core regulatory molecules, including p62, ROS, HIF-1α, and p53, serve as central hubs through which autophagy integrates and coordinates multiple hallmark categories. These nodes receive inputs from autophagic activity and, in turn, regulate downstream effectors across proliferative, dissemination, stress and plasticity, and microenvironmental and immune hallmarks. The interconnected network highlights the multifunctional role of these molecules in translating autophagic signals into context dependent hallmark outcomes.

Together, autophagy does not regulate individual hallmarks in isolation but instead creates a web of bidirectional crosstalk, and any therapeutic manipulation of autophagy is unlikely to produce a single, isolated effect; instead, it will ripple across the entire hallmark network in a context-dependent manner (Figure 3).

## 3. Autophagy-Targeted Therapies in Cancer

### 3.1 Pharmacological Modulators of Autophagy

#### 3.1.1 Core autophagy modulators

Core autophagy regulators target typical autophagy mechanisms such as autophagy initiation or phagophore nucleation levels (Table 1). A representative strategy in this category is to suppress autophagy initiation through disruption of the Beclin-1–VPS34 machinery. Spautin-1 most directly rewires the proliferative hallmarks and the stress and plasticity hallmarks. In preclinical models, spautin-1, a USP10/USP13 inhibitor, promotes Beclin-1 ubiquitination and degradation, destabilizes the VPS34–Beclin-1–ATG14 initiation complex, and thereby suppresses autophagy initiation 172. Under these conditions, reduced MEK/ERK–Cyclin D1 activity together with activation of MKK4/JNK/Bax has been observed, and this shift is associated with apoptosis and G1–S arrest, suggesting restored sensitivity to anti-proliferative cues in specific settings 173. In hematologic malignancy models, spautin-1 also reduces AKT Ser473 phosphorylation, activates GSK3β, decreases Mcl-1/Bcl-2, and enhances imatinib-induced apoptosis; in parallel, downregulation of GLUT1 increases cell death during glucose deprivation or ER stress 174. These findings indicate that direct inhibition of core autophagy machinery can weaken proliferative fitness not by directly shutting off a single oncogenic driver, but by removing the cytoprotective buffering that allows tumor cells to tolerate growth-associated stress. Besides, autophagy supports DNA-damage control, ROS buffering, and metabolic adaptation under replication or genotoxic stress, direct inhibition of the Beclin-1–VPS34 axis may expose tumor cells to a broader failure of adaptive homeostasis. In model systems, inhibition of the USP10/USP13 axis has also been linked to impaired DNA-damage responses, and spautin-1 can enhance DNA damage and sensitize cells to agents such as cisplatin 175. Accordingly, the therapeutic significance of this class lies not only in growth suppression, but also in restricting the stress tolerance and phenotypic flexibility that help tumor cells survive chemotherapy, nutrient limitation, or oxidative injury.

VPS34-driven autophagy is a typical adaptive mechanism of tumor cells to cope with amino acid/glucose deficiency, therapeutic pressure, and microenvironmental stress 176. VPS34-directed inhibition impairs autophagy-dependent metabolic adaptation and injury tolerance by directly impairing autophagosome nucleation. It has been reported that the Vps34 selective inhibitor SB02024 can block autophagy and increase the sensitivity of breast cancer cells to sunitinib/erlotinib 177. Furthermore, the Vps34 inhibitor SAR405 has been reported to increase intratumoral T cell infiltration and enhance anti-PD-1/PD-L1 activity in melanoma and colorectal cancer models. And autophagy inhibition of SAR405 leads to the formation of pTBK1-p62 aggregates, leading to the rearrangement of immune signals in different TNBC cells 178.

#### 3.1.2 Lysosome-targeting modulators

Lysosome-targeted modulators act downstream of core autophagy machinery and include drugs that disrupt lysosomal acidification, cargo degradation, membrane trafficking, and endolysosomal homeostasis. Lysosome-targeted modulators significantly rewired hallmarks of proliferation, stress and plasticity, and microenvironmental and immune, as well as propagation.

A major therapeutic consequence of lysosomal disruption is the weakening of tumor-cell survival buffering under cytotoxic or metabolic stress. Chloroquine (CQ) and hydroxychloroquine (HCQ) act late in the autophagy pathway, primarily by alkalinizing lysosomes and impairing lysosomal hydrolases, which leads to LC3-II and p62 accumulation and defective cargo degradation. In certain contexts and at particular doses, these agents may also induce lysosomal membrane permeabilization (LMP), leading to cathepsin release and amplification of mitochondrial apoptosis through increased ROS, caspase-9/-3 activation, and reduced Bcl-2 expression 179. Lys05, a dimeric aminoquinoline with stronger lysosomal accumulation, inhibits autophagic flux more potently and suppresses tumor growth in vitro and in vivo more effectively than HCQ in preclinical studies, although it remains a lead/tool compound without clinical validation 180. Mechanistically, lysosomal dysfunction and substrate accumulation increase cancer-cell susceptibility to apoptosis triggered by DNA damage or oxidative stress, providing a rationale for radio- and chemosensitization. For example, when autophagy is inhibited, DNA lesions induced by agents such as temozolomide are less efficiently cleared, resulting in increased cell death and improved antitumor activity in model systems, although the extent of this benefit is strongly context- and schedule-dependent 181.

Beyond direct tumor-cell killing, lysosome-targeting agents can also reshape the dissemination hallmarks through vascular and endothelial mechanisms. At low doses, chloroquine (CQ) and hydroxychloroquine (HCQ) are generally not directly cytotoxic; instead, they impair lysosomal acidification and lysosomal function, thereby disrupting endothelial polarity and endothelial–pericyte coupling 182. In preclinical models, these changes can reduce abnormal vascular permeability and improve perfusion and oxygenation, suggesting a process of vascular normalization rather than simple vessel ablation. In breast cancer and melanoma models, low-dose CQ has been reported to improve vascular architecture and perfusion, enabling deeper penetration of radiotherapy and chemotherapy while reducing microvessel density and enhancing treatment sensitivity 183. Overall, the available evidence supports the idea that targeting the autophagy–lysosome system can functionally normalize tumor vasculature, but these effects are strongly context- and schedule-dependent and may involve lysosomal mechanisms that are not strictly autophagy dependent 184.

Lysosome-targeting modulators are also highly relevant to the stress and plasticity hallmarks, particularly when tumor cells rely on autophagy–lysosome function to tolerate genotoxic stress or maintain stem-like survival states. As inhibitors of autophagic flux, chloroquine (CQ) and hydroxychloroquine (HCQ) prevent the clearance of DNA damage signals during chemotherapy and radiotherapy: γH2AX foci persist, RAD51 and BRCA1/2 assembly is impaired, and HR-mediated repair is compromised, ultimately resulting in defective double-strand break resolution 185. CQ and HCQ disrupt autophagy–lysosome function and weaken CSC- and EMT-associated phenotypes**.** More potent lysosomal agents such as Lys05, and where applicable DQ661, may intensify these effects by imposing stronger proteotoxic and metabolic stress on tumors that are highly dependent on lysosomal recycling.

A further therapeutic dimension of this class lies in its ability to recondition the tumor microenvironment and immune visibility of cancer cells. In preclinical models, agents targeting the autophagy–lysosome pathway, such as chloroquine (CQ), have shown improved tumor control when combined with single- or dual-agent immune checkpoint blockade (ICB), in part by limiting lysosome-dependent degradation of MHC-I and enhancing T-cell recognition; however, CQ also exerts autophagy-independent effects on lysosomal and endosomal trafficking 186. Similarly, melanoma studies indicate that low-dose CQ can repolarize tumor-associated macrophages (TAMs) toward an M1-like phenotype and reduce immunosuppressive as well as proinflammatory mediators, thereby modulating the inflammatory milieu; these effects likely involve both autophagy-dependent and autophagy-independent lysosomal or endosomal mechanisms and remain preclinical 187.

It should be noted that, although these lysosome-targeting agents are widely used as late-stage autophagy inhibitors, their effects are not limited to autophagic flux blockade. More precisely, they disrupt lysosomal acidification, cargo degradation, membrane trafficking, and endolysosomal homeostasis, with inhibition of autophagic flux representing one important consequence rather than the sole pharmacologic effect. Accordingly, their antitumor activities should not be uniformly attributed to autophagy blockade. In some contexts, such as impaired damage clearance and sensitization to cytotoxic stress, an autophagy-dependent component is likely to be important. In others, including vascular normalization, altered MHC-I turnover, and macrophage polarization, the observed phenotypes may more plausibly reflect mixed perturbations of lysosomal, endolysosomal, and autophagy-related pathways that are not strictly autophagy dependent 187,188.

#### 3.1.3 Upstream pathway modulators

Upstream pathway modulators do not primarily target core autophagy machinery or lysosomal execution steps. Instead, they act through broader nutrient sensing, metabolic or transcriptional regulatory network, with autophagy reprogramming emerging as an important downstream outcome rather than the sole initiating event. Its primary therapeutic effects include inhibiting anabolic growth, rewiring metabolic adaptations, and reshaping stress-responsive cellular state programs.

A major subgroup within this class includes mTOR-directed agents that suppress growth signaling while secondarily re-engaging autophagy. Rapamycin and its analogs, including everolimus and temsirolimus, allosterically inhibit mTORC1, rapidly suppress S6K1, and only partially restrain 4E-BP1 phosphorylation, thereby attenuating cap-dependent translation and growth signals 189. At the same time, release of ULK1/ATG13 and TFEB from mTORC1-mediated repression re-engages autophagy and lysosomal gene programs, lowering anabolic demand and producing a dual effect of growth suppression plus autophagy induction 190. Because mTORC1 also feeds into HIF-1α/VEGF signaling, its inhibition may additionally blunt angiogenic drive in some preclinical settings, although the magnitude of this effect is context dependent 191. Clinically, rapalogs have demonstrated benefit in selected tumor types: temsirolimus improves overall survival in metastatic renal-cell carcinoma (NCT00065468), and everolimus is approved for RCC and HR-positive breast cancer (NCT00410124; NCT00863655); however, feedback activation of PI3K/AKT and incomplete blockade of mTORC2 remain important resistance liabilities. In summary, rapalogs influence proliferation directly by inhibiting anabolic growth and cell cycle supporting translation programs, while their induction of autophagy represents a secondary adaptive response that can relieve stress or become cytoprotective depending on the situation.

A second major subgroup comprises metabolic regulators that secondarily engage autophagy through AMPK–mTOR or related axes. Metformin primarily targets mitochondrial complex I; this lowers ATP, elevates AMP, and activates AMP-activated protein kinase (AMPK), leading to Raptor phosphorylation, suppression of mTOR complex 1 (mTORC1), relief of ULK1 inhibition, and initiation of autophagy. In parallel, glycolysis-driven anabolism is curtailed, diminishing the proliferative advantage under high-glucose conditions and shifting metabolism toward catabolism 192. In breast and pancreatic cancer models, metformin has been reported to induce mitophagy and increase ROS, producing energetic stress and enhancing proapoptotic signaling; accordingly, AMPK–mTOR–autophagy-mediated rewiring may interrupt tumor metabolic programming in preclinical settings 193. Resveratrol remodels metabolism through SIRT1–AMPK and ROS–p53–DRAM signaling: activation of SIRT1/AMPK inhibits mTOR, promotes mitochondrial biogenesis, and increases autophagy, whereas p53 acetylation and Ser15 phosphorylation can promote DRAM-dependent autophagy that cooperates with apoptosis. At the same time, resveratrol suppresses glycolytic enzyme expression and GLUT1 and reduces lactate production, thereby attenuating the HIF-1α-dependent glycolytic bias 194. In NSCLC and other models, these effects are associated with activation of the NGFR–AMPK–mTOR axis, upregulation of autophagy markers such as LC3-II and BECN1, and reduced proliferation; where measured, flux readouts are consistent with increased autophagy 195. Overall, the impact of metformin and resveratrol on metabolic reprogramming is context-dependent and influenced by dose and schedule, with autophagy-independent mechanisms likely contributing alongside the AMPK–mTOR pathway. Similarly, Polyphyllin VI has been reported to induce apoptotic and autophagic cell death in non-small cell lung cancer through ROS-triggered mTOR signaling 196. These drugs influence tumor stress and plasticity characteristics, particularly metabolic reprogramming and adaptive stress responses, while their antiproliferative effects are seen as downstream consequences of altered energetic and biosynthetic status.

Autophagy reprogramming by upstream modulators may also intersect with nonmutational epigenetic adaptation. Spermidine, a small-molecule autophagy activator, inhibits EP300 and cooperates with AMPK, thereby inducing autophagy while reducing histone acetylation, highlighting the tractability of nonmutational epigenetic reprogramming in preclinical systems 197. These findings suggest that autophagy is not merely a catabolic stress-response pathway, but is functionally coupled to chromatin-associated adaptive programs that help tumor cells reconfigure transcriptional states under stress.

#### 3.1.4 Indirect autophagy-related modulators

Indirect autophagy-related modulators, first perturb broader cellular systems such as actin dynamics, ion homeostasis, vesicle trafficking, or organelle stress, with autophagy-related consequences emerging as secondary intersection points rather than initiating pharmacological events.

A representative example is cytochalasin E (CE), an actin polymerization inhibitor, which directly disrupts F-actin dynamics and blocks lamellipodia and filopodium formation, thereby impairing cancer cell movement 198. In addition to this direct effect on migratory structures, CE has also been reported to indirectly suppress autophagic flux by hindering cytoskeleton-dependent autophagosome transport and autophagosome–lysosome fusion, leading to accumulation of dysfunctional mitochondria and increased ROS 199. Consistent with these effects, CE reduce invasion-related phenotypes in preclinical systems by dismantling F-actin-dependent protrusions and modulating epithelial–mesenchymal transition programs, including reduction of mesenchymal markers and partial restoration of epithelial features. Unlike CQ and HCQ, which primarily act at the lysosomal degradation stage of autophagy, CE simultaneously disrupts migratory architecture and attenuates autophagic flux 200. However, CE remains a tool or preclinical compound with indirect effects on autophagy and limited translational feasibility.

A second subgroup within this category includes agents that perturb autophagy-related survival indirectly through ion homeostasis or endolysosomal trafficking. The ionophore salinomycin impairs autophagosome processing in acidic microenvironments, reducing breast CSC maintenance, whereas mefloquine perturbs endosome–lysosome trafficking, for example, through effects on RAB5/7-associated pathways, thereby disrupting the endocytosis–autophagy interface required for CSC survival and vesicular transport 201. Together, these studies indicate that the autophagy–lysosome network is not merely a passive buffering system, but an active determinant of whether tumor cells can sustain stem-like properties, adapt to hostile niches, and transition between epithelial and mesenchymal states.

Taken together, indirect autophagy-related regulators limit dispersal-related traits by dismantling migratory and invasive structures and limit stress adaptation or stemness-related survival states. Among these drugs, autophagy may be better understood as a functionally cross-functional process, presenting potential for combination therapy, rather than as a primary molecular target.

### 3.2 Combination Strategies

Pharmacological modulation of autophagy is rarely used as monotherapy; rather, it is most effective when used as a sensitizing strategy in combination with other therapeutic modalities. Increasing preclinical and clinical evidence has demonstrated that autophagy modulators enhance the efficacy of chemotherapy, radiotherapy, targeted agents, and immunotherapies by dismantling tumor dependencies at the hallmark level 202. Table 2 summarizes key clinical and translational combinations.

#### 3.2.1 Targeting Proliferative Signaling and Metabolic Reprogramming

mTOR inhibitors (rapalogs) and metabolic stressors frequently induce compensatory autophagy via ULK1 activation and nutrient-sensing feedback loops 203. When combined with late-step autophagy inhibitors such as CQ, HCQ, or next-generation lysosomotropic agents (ROC-325, Lys05), this survival mechanism is blocked, leading to sustained suppression of cap-dependent translation, depletion of recycled substrates, and collapse of mitochondrial metabolism 204. RAS-driven tumor models treated with trametinib plus HCQ have demonstrated regression, highlighting that cotargeting MAPK signaling and autophagic recycling disrupts both the maintenance of proliferative signaling and metabolic reprogramming hallmarks more effectively than either agent alone 205.

#### 3.2.2 Overcoming Resistance to Cell Death

Genotoxic therapies such as radiotherapy, temozolomide, cisplatin, and paclitaxel commonly induce protective autophagy, thereby enabling the clearance of damaged organelles and facilitating DNA repair. Pharmacologic inhibition with CQ, Lys05, or ROC-325 converts this cytoprotective response into apoptosis or necrosis by promoting autophagosome accumulation, reactive oxygen species overload, and caspase activation 204,206,207. Clinical trials combining HCQ with temozolomide and radiotherapy in glioblastoma have reported extended median overall survival (15.6 months) and pharmacodynamic confirmation of autophagy blockade (LC3-II and p62 accumulation) (NCT00486603). In cisplatin-resistant ovarian xenografts, CQ overcomes multidrug resistance by blocking autophagosome–lysosome fusion and overwhelming the capacity for DNA repair 208.

#### 3.2.3 Restoring Immune Visibility

Autophagy contributes to immune evasion by mediating lysosomal degradation of MHC-I and limiting antigen presentation. Inhibition with CQ-class agents or VPS34 inhibitors restores surface MHC-I, enhances dendritic cell priming, and augments the response to immune checkpoint blockade 209. In preclinical models of pancreatic ductal adenocarcinoma, combining autophagy inhibition with PD-1/CTLA-4 antibodies significantly increased CD8^+^ T-cell infiltration, reprogrammed tumor-associated macrophages, and suppressed metastasis. These effects directly counter two key hallmarks of cancer: evasion of immune destruction and activation of invasion and metastasis 210. Autophagy-modulating combinations with immunotherapy are also being explored clinically, including cobimetinib + atezolizumab + HCQ in KRAS-mutant advanced malignancies, HCQ with nivolumab ± ipilimumab in melanoma, and avelumab-containing perioperative regimens in pancreatic cancer (NCT04214418; NCT04464759; NCT03344172).

#### 3.2.4 Amplifying Genomic Instability and the Stress Response

Autophagy supplies nucleotides and removes damaged mitochondria during genotoxic stress 211. Its inhibition during DNA-damaging therapy increases the lesion burden, induces mitotic catastrophe, and increases apoptosis. Compared with monotherapy, the combination of PARP inhibitors, radiotherapy, or platinum agents with late-stage autophagy blockade results in superior tumor regression 212. Dual-function agents such as LS-110 integrate DNA damage induction and autophagy inhibition, further pushing tumor cells beyond their repair capacity and exploiting the Genome Instability hallmark for therapeutic gain 213.

Taken together, combination strategies that integrate autophagy modulation with chemotherapy, radiotherapy, targeted therapy, and immunotherapy produce hallmark-level synergy (Table 2).

### 3.3 Clinical Trials and Challenges

#### 3.3.1 Radiotherapy Sensitization

Clinical trials investigating the use of autophagy inhibitors as radiosensitizers have focused predominantly on glioblastoma and brain metastases (NCT00486603; NCT01602588; NCT01894633; NCT02378532; NCT02432417). In a phase I/II trial involving newly diagnosed glioblastoma patients, HCQ in combination with radiotherapy and temozolomide was administered up to a maximum tolerated dose of 600 mg/day, with autophagy inhibition confirmed by LC3-II accumulation, resulting in a median overall survival of approximately 15.6 months (NCT00486603). However, in a randomized phase II study in elderly patients with newly diagnosed high-grade glioma, HCQ plus short-course radiotherapy was feasible and safe but did not improve survival compared with radiotherapy alone (NCT01602588). In patients with brain metastases, CQ combined with whole-brain radiotherapy achieved high rates of intracranial disease control, with minimal severe toxicity observed (NCT01894633). Smaller trials, including randomized studies in younger glioblastoma cohorts, have reported survival extensions of up to 33 months following the addition of CQ (NCT00224978). Although safety and feasibility have been consistently demonstrated, survival benefits have varied, potentially due to tumor heterogeneity, differences in trial design, and patient selection criteria, underscoring the need for biomarker-driven approaches and standardized protocols to optimize the therapeutic efficacy of autophagy inhibition in combination with radiotherapy.

#### 3.3.2 Chemotherapy Sensitization and MDR

The modulation of autophagy has been recognized as a strategic approach to sensitize tumors to chemotherapy and overcome MDR. Clinical trials have focused primarily on the repurposing of CQ and HCQ, with encouraging efficacy signals reported in NSCLC, pancreatic cancer, colorectal cancer, and hepatocellular carcinoma (NCT01026844; NCT00977470; NCT01506973; NCT01978184; NCT01206530; NCT03037437). Notably, HCQ combined with erlotinib was well tolerated and demonstrated potential efficacy in treating epidermal growth factor receptor-tyrosine kinase inhibitor (EGFR-TKI)–refractory NSCLC (NCT01026844; NCT00977470). HCQ-based regimens combined with sorafenib or FOLFOX/bevacizumab have also been explored clinically (NCT03037437; NCT01206530). In pancreatic cancer, HCQ has been tested with gemcitabine/nab-paclitaxel in both metastatic and preoperative settings (NCT01506973; NCT01978184). Safingol, an agent that targets sphingolipid metabolism, has been shown to enhance cytotoxicity in combination with cisplatin; however, hepatotoxicity remains a significant concern (NCT00084812). MK-2206, an allosteric AKT inhibitor, is being evaluated in early-phase clinical trials and is proposed to restore chemosensitivity by suppressing survival signaling pathways (NCT01480154). In addition to these agents, emerging autophagy inhibitors (e.g., ROC-325 and Lys05) and nanocarrier systems designed for codelivery are under preclinical investigation 214. Despite early promise, clinical translation has remained limited by toxicity, tumor heterogeneity, and an incomplete understanding of the underlying mechanisms; nevertheless, targeting autophagy remains a compelling strategy when paired with rational combinations and biomarker-guided selection.

#### 3.3.3 Targeted Therapy and Metabolic Modulation

Autophagy dependency has increasingly been recognized as a metabolic vulnerability in tumors driven by hyperactivation of the RTK/RAS/PI3K/mTOR pathways. Rather than targeting autophagy in isolation, recent clinical efforts have focused on coinhibiting convergent signaling and metabolic nodes. In renal cell carcinoma, HCQ has been combined with mTOR inhibitors such as everolimus and temsirolimus (NCT01510119; NCT00909831). In colorectal cancer, HCQ has been added to FOLFOX/bevacizumab (NCT01206530), and in metastatic BRAF-mutant colorectal cancer it is now being tested with encorafenib plus cetuximab or panitumumab (NCT05576896). In BRAF-mutant melanoma, dual targeting of MAPK signaling and autophagy has been implemented with dabrafenib, trametinib, and HCQ (NCT02257424), whereas in pancreatic cancer trametinib plus HCQ is under evaluation (NCT03825289). Broader metabolism/autophagy-stress combinations that incorporate sirolimus, metformin, dasatinib, nelfinavir, and HCQ are also being tested in advanced solid tumors within the COAST platform (NCT05036226). In glioblastoma, CQ has similarly been repurposed alongside radiochemotherapy in newly diagnosed disease (NCT02378532; NCT02432417). These trials collectively reflect a conceptual shift: autophagy is being leveraged as a signaling and stress-adaptation scaffold whose therapeutic value emerges most clearly when paired with pathway-directed combinations.

#### 3.3.4 Combining Immunotherapy

**Figure 4:**
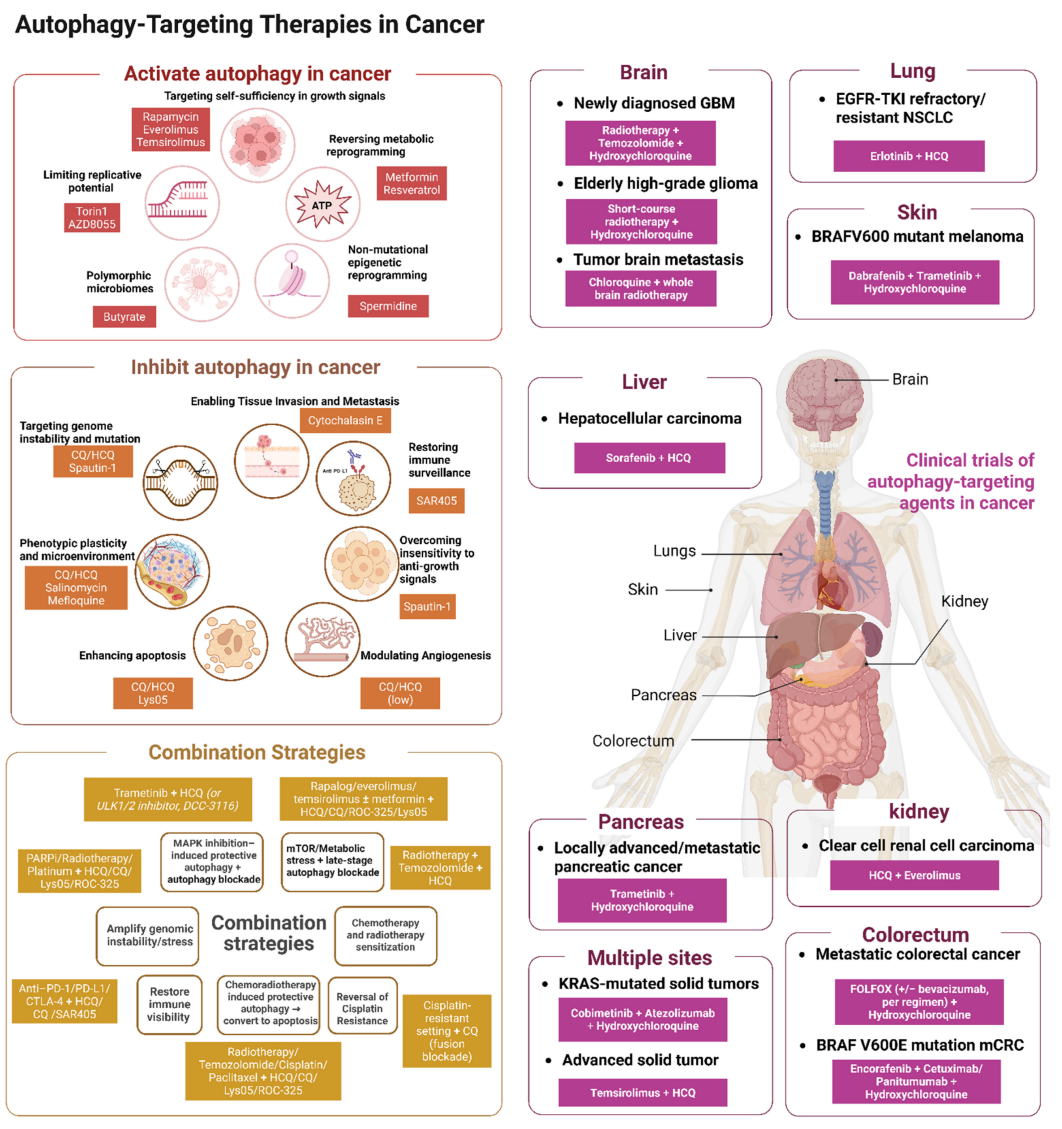
** Autophagy-Targeting Therapies in Cancer.** The left side illustrates preclinical drug research that modulates tumor hallmarks by targeting autophagy, including activators, inhibitors, and combination therapies, whereas the right side lists compounds undergoing clinical trials for cancer treatment that target autophagy.

Clinical trials investigating the combination of autophagy modulators with immunotherapy have emerged as promising strategies across multiple cancers. In advanced solid tumors harboring KRAS mutations, the cobimetinib + atezolizumab + HCQ combination has entered phase I/II evaluation (NCT04214418). In melanoma, HCQ has been investigated in combination with nivolumab alone or nivolumab/ipilimumab, primarily to assess safety, tolerability, and preliminary efficacy (NCT04464759). In resectable pancreatic cancer, avelumab has also been added to gemcitabine/nab-paclitaxel plus HCQ in a randomized preoperative design (NCT03344172). Other agents, including pevonedistat and MK-2206, are currently under investigation across diverse tumor types, but conclusive data on their clinical benefits remain limited (NCT04891755; NCT01480154). These trials collectively suggest that the modulation of autophagy may potentiate immunotherapy; however, challenges remain in optimizing drug selection and elucidating the mechanisms of synergy (Figure 4, Table 2).

#### 3.3.5 Limitations: Context-dependent Efficacy, Toxicity, and Biomarker Development

The clinical translation of autophagy modulation has been constrained by pronounced context dependence, reflecting the intrinsic plasticity of both cancer cells and the autophagy pathway. Across clinical trials, efficacy varies not only by tumor type but also by molecular subtype and treatment setting. For example, survival signals were more encouraging in some glioblastoma cohorts receiving chloroquine-based radiosensitization (NCT00224978), whereas HCQ added to short-course radiotherapy did not improve survival in elderly patients (NCT01602588). Comparable heterogeneity is also evident in pancreatic cancer, where preoperative or randomized HCQ-containing regimens yielded encouraging signals in selected settings (NCT01978184), yet metastatic disease did not show improved 1-year overall survival with gemcitabine/nab-paclitaxel plus HCQ (NCT01506973). Toxicity likewise limits scalability: although HCQ has a relatively acceptable safety profile, dose escalation and chronic use remain constrained, and newer combinations such as safingol/cisplatin (NCT00084812) or MK-2206/HCQ (NCT01480154) still require careful therapeutic-window definition. Compounding these challenges is the lack of reliable biomarkers that quantify autophagic flux or predict response in real time; thus, general toxicity/biomarker citations in this subsection should largely remain as conventional references rather than being forcibly replaced by NCT identifiers.

## 4. Conclusions and Future Perspectives

Autophagy plays a pivotal role in maintaining cellular homeostasis and quality control by degrading damaged or aged organelles and misfolded proteins 12. It performs multiple physiological functions in organisms, for example, it breaks down macromolecules such as proteins, nucleic acids, and carbohydrates to supply nutrients and increase energy metabolism 215; Moreover, autophagy can engulf and degrade invading bacteria, participate in antigen presentation, and subsequently regulate immune responses 9. These autophagy-regulated physiological processes are closely associated with the hallmarks of cancer, establishing autophagy as a core enabler of multidimensional biological capabilities in tumors. Rather than uniformly promoting or suppressing tumorigenesis, autophagy acts as a critical “adaptive toolbox” that tumor cells exploit to acquire and sustain multiple hallmark traits. Its specific functions are highly dependent on the tumor type, developmental stage, and microenvironmental context 216. During the early stages of tumorigenesis or in precancerous lesions, autophagy functions primarily as a tumor-suppressive mechanism. It maintains genomic stability by clearing damaged mitochondria and misfolded protein aggregates, thereby preventing ROS-induced DNA damage and indirectly suppressing uncontrolled proliferation 217,218. However, in established tumors, autophagy often plays a prosurvival role. Tumor cells exploit autophagy to recycle nutrients and provide essential building blocks, including amino acids and nucleotides, for sustained biosynthesis, while simultaneously eliminating toxic protein aggregates and damaged organelles 219. This process enhances resistance to apoptosis induced by chemotherapy, radiotherapy, or targeted therapy 220. Consequently, autophagy represents a key mechanism underlying various forms of treatment resistance.

The context-dependent role of autophagy in tumor biology is attributable mainly to tumor heterogeneity. Even within the same tumor type, significant heterogeneity can be observed across different developmental stages and even within the same progressive phase 221. Notably, although all tumors acquire specific hallmark capabilities during their evolution, including resistance to apoptosis, sustained angiogenesis, and limited replicative potential, considerable variation exists among different tumors with respect to the timing, order of acquisition, and degree of dependence on these features 222. It is essential to delineate a tumor's genetic background to determine its reliance on specific hallmarks, thereby facilitating the design of targeted therapeutic strategies that exploit context-specific autophagic functions. Therefore, based on the hallmark-level crosstalk mechanisms synthesized above, we propose a conceptual hypothesis: the therapeutic outcome of autophagy modulation, be it inhibition or activation, is determined by the alignment between a tumor’s hallmark dependency landscape and its basal autophagic flux relative to the optimal range for each hallmark category. For instance, in tumors that display strong dependency on proliferation and dissemination hallmarks, such as KRAS mutant pancreatic cancer, elevated autophagic flux is required to meet biosynthetic and energetic demands. In this setting, pharmacological inhibition of autophagy is predicted to suppress flux below the optimal range, thereby depriving tumor cells of metabolic support and restraining proliferation and invasion 223,224. Conversely, tumors that rely heavily on immune evasion and stress adaptation hallmarks, such as melanoma and squamous cell carcinoma, often exhibit autophagic insufficiency when treated with autophagy inhibitors, leading to p62 accumulation that actively drives these pathways. In these tumors, impaired autophagic flux leads to p62 accumulation, which activates NF-κB via JNK signaling, upregulating inflammatory cytokines such as IL-8 and HIF-1α. Furthermore, p62 serves as a selective autophagy receptor that directly binds PD-L1 and promotes its autophagic degradation; p62 accumulation therefore stabilizes PD-L1, contributing to immune evasion. Autophagy activation is therefore predicted to restore flux, degrade p62, and attenuate both inflammation and immune suppression 225. This conceptual framework provides a practical roadmap for future clinical decision-making. It suggests that patient selection for autophagy-targeted therapies should be guided by two complementary parameters: hallmark dependency signatures and autophagic flux status. Together, these parameters offer actionable guidance for future trial design and biomarker-driven patient stratification.

Autophagy exhibits a dualistic nature in cancer, functioning as both a tumor suppressor and a tumor promoter. This dichotomy necessitates a critical reassessment of how pharmacological modulators of autophagy are deployed therapeutically 226,227. However, the regulatory role of autophagy in tumors is not a simple matter of activation or inhibition, but rather a context-dependent process that is precisely regulated by the specific tumor microenvironment. This necessitates an in-depth understanding of the specific relationships between autophagy and the hallmarks of cancer, as well as the development of precise methods for monitoring and modulating autophagic flux. Current methods for monitoring autophagic flux, including transmission electron microscopy, tissue sectioning, Western blotting, and probe-based systems such as mCherry-GFP-LC3, have enabled the assessment of autophagic flux in specific mouse tissues 228. However, these approaches primarily provide endpoint measurements at a single time point and lack the capacity for real-time monitoring. Given that autophagic activity in tumors dynamically fluctuates throughout cancer progression and in response to therapy, the ability to monitor autophagic flux in real time is of critical importance. Nevertheless, several bottlenecks hinder the clinical implementation of real-time autophagic flux monitoring in tumor tissues. For instance, tumor samples can only be obtained through invasive procedures such as biopsy or surgical resection; repeated sampling from the same patient is neither practical nor ethically justifiable. Moreover, these specimens represent static snapshots, which inevitably introduce discrepancies when used to infer the dynamic changes in autophagic flux occurring within the tumor. In the future, achieving real-time detection of tumor autophagic flux may require breakthroughs in several directions. First, the identification of an ideal biomarker that can be detected non-invasively in peripheral blood samples. Second, the development of novel probes that can be integrated with existing human imaging technologies to enable real-time visualization of autophagic processes. Nevertheless, given the current state of technological development, the path toward real-time monitoring of autophagic flux remains fraught with challenges, and considerable progress is still needed before clinical application becomes feasible.

Currently, significant progress has been made in the development of small-molecule drugs that target autophagy for cancer therapy; however, numerous challenges remain. The primary issues lie in the specificity and safety of these agents: many commonly used autophagy inhibitors exhibit broad target engagement rather than selectively acting on autophagy-related proteins, which may lead to off-target effects and severe adverse reactions. Furthermore, given the tissue-specific regulation of autophagy, systemic inhibition of autophagy may cause significant toxicity in normal tissues. This necessitates the precise modulation of autophagic activity within tumor sites, imposing stricter requirements not only on the autophagy-regulating capacity of the compounds but also on their pharmacokinetic properties, including tissue distribution, accumulation, and metabolism. Therefore, there is an urgent need to develop more selective small-molecule modulators that target key proteins or genes within the autophagy pathway to achieve precise intervention in autophagy. Concurrently, in-depth investigations and optimization of their pharmacokinetic profiles are essential. When necessary, targeted drug delivery systems can be employed to increase drug enrichment in tumor tissues and improve treatment specificity. Moreover, the limited therapeutic window of monotherapeutic autophagy modulators often stems from the dynamic and complex nature of autophagy itself, rendering it inherently challenging to modulate with high precision. To address this challenge, reducing the dose of single-agent therapy and combining it with chemotherapy, targeted therapy, or immunotherapy may have synergistic effects, enhance antitumor efficacy, and reduce both toxicity and the risk of drug resistance. Finally, the high heterogeneity of tumors necessitates systematic profiling of individual tumor biological features before treatment. Identifying the core biological hallmarks and associated signaling pathways that a cancer depends on will facilitate the development of small-molecule drugs that selectively modulate these pathways, thereby achieving enhanced efficacy and reduced toxicity. On this basis, mechanism-guided combination strategies that cotarget multiple core and emerging cancer hallmarks hold promise for delivering more effective and durable treatment options for cancer patients.

In this review, we integrated the evolving cancer hallmarks framework and proposed a mechanistic classification comprising four categories based on tumor initiation and progression. Within this framework, we systematically described the molecular mechanisms by which autophagy regulates these hallmarks and the crosstalk among them. These insights offer new perspectives on autophagy's role in tumor biology and its potential as a hallmark-informed therapeutic strategy. We also summarized autophagy-targeting small-molecule compounds and explained their effects on tumor biology through the lens of cancer hallmarks. Finally, we propose that the therapeutic outcome of autophagy modulation depends on the alignment between a tumor's hallmark dependency landscape and its basal autophagic flux relative to the optimal range for each hallmark category. By bridging the cancer hallmarks theory with autophagy biology, this approach may provide novel concepts and tools for current and future anticancer therapies.

## Figures and Tables

**Table 1:** Autophagy modulators: mechanisms and corresponding cancer hallmarks.

Type	Drug name	Primary target	Relationship to autophagy	Hallmarks	Mechanism	Clinical status
Core autophagy modulators	Spautin-1	USP10/USP13→Beclin-1-VPS34-ATG14 complex	Direct inhibition of autophagy initiation	Proliferative fitness; stress tolerance; phenotypic plasticity	Promotes Beclin-1 degradation, destabilizes the VPS34 initiation complex, suppresses cytoprotective buffering, and enhances apoptosis/stress sensitivity	Preclinical
	SAR405	VPS34 (PIK3C3)	Direct inhibition of autophagosome nucleation	Metabolic adaptation; damage tolerance; immune signaling	Impairs autophagy-dependent metabolic adaptation and damage tolerance; in selected models also alters immune signaling and enhances T-cell infiltration	Preclinical
	SB02024	VPS34 (PIK3C3)	Direct inhibition of autophagosome nucleation	Metabolic adaptation; therapy tolerance; proliferative fitness	Blocks autophagy and sensitizes tumor cells to targeted therapy, consistent with loss of stress buffering and therapy tolerance	Preclinical
Lysosome-targeting modulators	Chloroquine (CQ)	Lysosomal acidification / endolysosomal system	Blocks autophagic flux but also exerts broader lysosomal/endosomal effects	Apoptosis resistance; metabolic stress adaptation; immune evasion; vascular remodeling	Disrupts lysosomal acidification and cargo degradation; effects may reflect mixed autophagy-dependent and autophagy-independent mechanisms, including apoptosis sensitization, vascular normalization, altered MHC-I turnover, and TAM repolarization	Clinical trials / repurposed
	Hydroxychloroquine (HCQ)	Lysosomal acidification / endolysosomal system	Blocks autophagic flux but also exerts broader lysosomal/endosomal effects	Apoptosis resistance; metabolic stress adaptation; immune evasion; vascular remodeling	Similar to CQ, with a better tolerated clinical profile; widely used in combination trials as a lysosome-targeting autophagy modulator	Clinical trials / repurposed
	Lys05	Lysosome	Potent lysosomotropic inhibition of lysosomal function and autophagic flux	Apoptosis resistance; metabolic stress adaptation	Stronger lysosomal accumulation than CQ/HCQ; intensifies proteotoxic/metabolic stress and therapy sensitization	Preclinical
	DQ661	PPT1 / lysosomal function	Lysosomal inhibition with autophagy- and mTOR-related downstream consequences	Metabolic fitness; therapy tolerance; immune evasion	Disrupts lysosomal recycling and metabolic fitness in lysosome-dependent tumors	Preclinical
Upstream pathway modulators	Rapamycin	mTORC1	Indirect reprogramming of autophagy through upstream nutrient-sensing control	Anabolic growth signaling; cell-cycle support; stress adaptation	Suppresses anabolic growth signaling and translation; autophagy induction is a downstream adaptive consequence rather than the primary initiating event	FDA-approved
	Everolimus / Temsirolimus	mTORC1	Indirect reprogramming of autophagy through upstream nutrient-sensing control	Anabolic growth signaling; cell-cycle support; stress adaptation	Rapalogs restrain growth signaling and cell-cycle support while secondarily re-engaging autophagy/lysosomal programs	FDA-approved
	Torin1 / AZD8055	mTORC1/2	Indirect reprogramming of autophagy through broader mTOR blockade	Anabolic growth signaling; replication stress handling; metabolic adaptation	More completely suppress growth signaling than rapalogs and secondarily reactivate autophagy-linked stress handling programs	Preclinical
	Metformin	Mitochondrial complex I -> AMPK-mTOR	Indirect metabolic reprogramming with secondary autophagy engagement	Metabolic reprogramming; energetic stress adaptation; proliferative restraint	Imposes energetic stress, activates AMPK, suppresses mTORC1, and rewires tumor metabolic adaptation	Clinical trials / repurposed
	Resveratrol	SIRT1-AMPK / ROS-p53-DRAM	Indirect metabolic and stress-response reprogramming with secondary autophagy engagement	Metabolic reprogramming; oxidative stress responses; proliferative restraint	Rewires metabolism and stress signaling, with autophagy as one component of a broader adaptive response	Preclinical
	Spermidine	EP300 / AMPK	Indirect autophagy activation coupled to epigenetic adaptation	Epigenetic plasticity; adaptive transcriptional state control	Links autophagy induction to nonmutational epigenetic reprogramming and adaptive transcriptional state control	Preclinical
Indirect autophagy-related modulators	Cytochalasin E (CE)	Actin polymerization / cytoskeletal dynamics	Indirect intersection with autophagy via cytoskeleton-dependent autophagosome trafficking	Invasion; migration; EMT-associated plasticity	Disrupts migratory architecture and EMT-linked invasion programs; autophagy attenuation is contributory rather than primary	Preclinical
	Salinomycin	Ion homeostasis / acidic microenvironment stress	Indirect perturbation of autophagosome processing	CSC maintenance; niche adaptation; metastatic competence	Weakens CSC maintenance and stress-adaptive survival under hostile niches	Preclinical
	Mefloquine	Endosome-lysosome trafficking / RAB5/7-related pathways	Indirect perturbation of the endocytosis-autophagy interface	CSC-supporting survival; vesicular transport; metastatic competence	Disrupts vesicular transport and CSC-supporting survival programs rather than directly targeting canonical autophagy machinery	Preclinical

**Table 2:** Clinical landscape of autophagy modulators in cancer.

Clinical trials of autophagy modulators combined with radiotherapy					
Tumor Type	Agent	Phase	Key Finding	NCT/Ref.	Other
Newly diagnosed glioblastoma multiforme	HCQ + RT + TMZ	Phase I/II	MTD ~600 mg/day; autophagy inhibition confirmed; OS ~15.6 months Extended median OS (~24–33 months vs ~11 months) in CQ arms	24991840 8496728 NCT00486603	--
Elderly high-grade glioma	HCQ + RT	Phase II (RCT)	Safe, but final OS/PFS data not detailed publicly	32642699 NCT01602588	--
Brain metastases (solid tumors)	CQ + WBRT	Phase II pilot	High intracranial response, no severe toxicity	24187608	--
Clinical trials of autophagy modulators combined with MDR					
NSCLC (EGFR-mutant or -TKI resistant)	HCQ + Erlotinib	Phase I/II	HCQ was well tolerated and showed potential to enhance the response to erlotinib in cases of resistance.	NCT00977470	--
Solid tumors (various advanced)	Safingol + Cisplatin	Phase I	Safingol enhanced cisplatin cytotoxicity with manageable liver toxicity.	NCT00084812	--
Various solid tumors (with fenretinide)	Safingol + Fenretinide	Phase I	The combination was administered safely, supporting further evaluation of MDR-targeted therapies.	NCT01553071	--
Gastric/gastroesophageal junction cancer	MK-2206 (AKT inhibitor) single-agent or in combinations (e.g., trastuzumab, chemotherapy)	Phase II	MK-2206 showed modest activity and acceptable safety as monotherapy in gastric cancer. Combination therapy was tolerable, and suggested AKT pathway inhibition may reverse resistance.	NCT01260701 (gastric), NCT00963547 (combo), NCT01312753 (endometrial)	--
Clinical trials of autophagy modulators combined with targeted therapy and metabolic modulation					
Renal cell carcinoma	HCQ + Everolimus	Phase I/II	The combination was tolerable with early antitumor signals	NCT01510119	mTOR inhibitors + metabolic modulation enhancing autophagy & apoptosis
mCRC	HCQ + Sirolimus + Metformin + Dasatinib + Nelfinavir	Phase I/II	Evaluated metabolic cotargeting of autophagy; regimen tolerated	NCT05036226	
Breast/Endometrial/CRC	Metformin (monotherapy or adjuvant)	Phase III	Under investigation for impact on metabolic signaling and autophagy induction	NCT01101438 NCT02614339 NCT02065687	
Neuroblastoma/Cholangiocarcinoma	HCQ + Trametinib	Phase II	Disease control observed in RAS-mutant tumors supports metabolic-vulnerability targeting.	NCT03979651 NCT04566133	RAS/RAF/RTK-driven tumors leveraging autophagy dependency
BRAF-mutant CRC	HCQ + Encorafenib + Cetuximab or Panitumumab	Phase II	Safety and efficacy under evaluation; targets MAPK-autophagy axis	NCT05576896	
Hepatocellular carcinoma	Sorafenib + HCQ	Phase II	Under study for overcoming resistance and enhancing metabolic suppression	NCT03037437	
NSCLC	HCQ + Erlotinib	Phase I	HCQ safely combined with EGFR-TKI; signal of activity in resistant tumors	NCT00977470	Autophagy inhibition + EGFR/RTK inhibitors breaching proliferative and metabolic hallmarks
NSCLC (advanced)	HCQ + Paclitaxel + Carboplatin + Bevacizumab	Phase II	Multimodal therapy, including autophagy blockade, showed feasibility	NCT01649947	
Glioblastoma	CQ + Radiotherapy + Temozolomide	Phase I	CQ enhanced treatment response in resistant glioma models	NCT02378532	Autophagy inhibition undermines energy metabolism in hypoxic or therapy-resistant clones.
Glioma/melanoma	Temsirolimus + HCQ	Phase I/II	Pharmacodynamic evidence of autophagy suppression	36678822	
Clinical trials of autophagy modulators combined with immunotherapy					
Hepatocellular carcinoma	GNS561 + Atezolizumab + Bevacizumab	Phase II	The safety and efficacy were evaluated, and the initial results showed that the combination therapy had potential.	NCT05448677	--
NSCLC	HCQ + bevacizumab	Phase I	To evaluate the safety of HCQ as an autophagy inhibitor in combination with monoclonal antibodies	NCT01649947	--
Multiple tumor types	Pevonedistat + Immunotherapy	--	Evaluate the efficacy of Pevonedistat combined with immunotherapy	PMID: 8946974	--
Multiple tumor types	Safingol + Chemotherapy	Phase I	The assessment of safety and efficacy is still in its early stages	NCT00084812	--
Multiple tumor types	MK-2206 + Chemotherapy/Targeted therapy	Phase II	The combined therapeutic effect is being evaluated	NCT01658943	--

## Abbreviations

AKT: Protein kinase B
AMPK: AMP-activated protein kinase
ATG: Autophagy-related gene/protein
BAK: Bcl-2 antagonist killer
BAX: Bcl-2-associated X protein
Bcl-2: B-cell lymphoma 2
CMA: Chaperone-mediated autophagy
CQ: Chloroquine
DDR: DNA damage response
EMT: Epithelial–mesenchymal transition
ERK: Extracellular signal-regulated kinase
ETC: Electron transport chain
FIP200: FAK family-interacting protein of 200 kDa
FUNDC1: FUN14 domain-containing protein 1
GLUT1: Glucose transporter 1
HCQ: Hydroxychloroquine
HIF-1α: Hypoxia-inducible factor-1 alpha
ICB: Immune checkpoint blockade
JNK: c-Jun N-terminal kinase
LC3-II: Phosphatidylethanolamine-conjugated LC3
MAPK: Mitogen-activated protein kinase
Mcl-1: Myeloid cell leukemia 1
MFN2: Mitofusin 2
MMPs: Matrix metalloproteinases
MOMP: Mitochondrial outer membrane permeabilization
mTOR: Mechanistic target of rapamycin
NB: Neuroblastoma
NF-κB: Nuclear factor kappa-light-chain-enhancer of activated B cells
NSCLC: Non-small-cell lung cancer
OS: Overall survival
PD-1: Programmed cell death protein 1
PD-L1: Programmed death-ligand 1
PFS: Progression-free survival
PI3K/PI3KC3: Phosphatidylinositol 3-kinase/class III PI3K complex
PINK1: PTEN-induced putative kinase 1
PTEN: Phosphatase and tensin homolog
p53: Tumor protein 53
ROS: Reactive oxygen species
SIRT1: Sirtuin 1
SQSTM1/p62: Sequestosome 1
STAT3: Signal transducer and activator of transcription 3
TBK1: TANK-binding kinase 1
TME: Tumor microenvironment
TMZ: Temozolomide
TRAIL: TNF-related apoptosis-inducing ligand
TP53: Tumor protein p53
ULK1: Unc-51-like kinase 1
USP: Ubiquitin-specific protease
V-ATPase: Vacuolar-type H⁺-ATPase
VDAC1: Voltage-dependent anion channel 1
VEGF: Vascular endothelial growth factor
